# Nature-based solutions efficiency evaluation against natural hazards: Modelling methods, advantages and limitations

**DOI:** 10.1016/j.scitotenv.2021.147058

**Published:** 2021-08-25

**Authors:** Prashant Kumar, Sisay E. Debele, Jeetendra Sahani, Nidhi Rawat, Belen Marti-Cardona, Silvia Maria Alfieri, Bidroha Basu, Arunima Sarkar Basu, Paul Bowyer, Nikos Charizopoulos, Glauco Gallotti, Juvonen Jaakko, Laura S. Leo, Michael Loupis, Massimo Menenti, Slobodan B. Mickovski, Seung-Jae Mun, Alejandro Gonzalez-Ollauri, Jan Pfeiffer, Francesco Pilla, Julius Pröll, Martin Rutzinger, Marco Antonio Santo, Srikanta Sannigrahi, Christos Spyrou, Heikki Tuomenvirta, Thomas Zieher

**Affiliations:** a Global Centre for Clean Air Research (GCARE), Department of Civil and Environmental Engineering, Faculty of Engineering and Physical Sciences, University of Surrey, Guildford GU2 7XH, United Kingdom; b Department of Civil, Structural & Environmental Engineering, School of Engineering, Trinity College Dublin, Dublin, Ireland; c Department of Geoscience and Remote Sensing, Delft University of Technology, Delft, the Netherlands; d School of Architecture, Planning and Environmental Policy, University College Dublin, Dublin, Ireland; e Climate Service Center Germany (GERICS), Helmholtz-Zentrum Hereon, Hamburg, Germany; f Agricultural University of Athens, Laboratory of Mineralogy-Geology, Iera Odos 75, 118 55 Athens, Greece; g Region of Sterea Ellada, Kalivion 2, 351 32 Lamia, Greece; h Department of Physics and Astronomy (DIFA), Alma Mater Studiorum-University of Bologna, Bologna, Italy; i Finnish Meteorological Institute, Erik Palménin Aukio 1, 00560 Helsinki, Finland; j Innovative Technologies Center S.A., Alketou Str. 25, 11633 Athens, Greece; k National & Kapodistrian University of Athens, Psachna 34400, Greece; l Aerospace Information Research Institute, Chinese Academy of Sciences, Beijing, China; m The Built Environment Asset Management Research Centre, Glasgow Caledonian University, G4 0BA Glasgow, Scotland, United Kingdom; n Institute for Interdisciplinary Mountain Research, Austrian Academy of Sciences, Innsbruck, Austria; o Institute of Geography, University of Innsbruck, Innsbruck, Austria; p Institute for Astronomy, Astrophysics, Space Applications and Remote Sensing (IAASARS), National Observatory of Athens, 15236 Athens, Greece

**Keywords:** 1D, one-dimensional, 2D, two-dimensional, 3D, three-dimensional, ACRU, Agricultural Catchments Research Unit, ADCIRC, Advanced Circulation Model for Shelves, Coastal Seas, and Estuaries, ArcGIS, Geographic Information System, BE-HAM, Building Envelope Heat and Moisture, BROOK90, Physically-Based Hydrological Model, CBA, Cost Benefit Analysis, CCA, climate change adaptation, CFD, computational fluid dynamics, CO_2_, carbon dioxide, DBH, Diameter Breast Height, DEM, digital elevation model, DRR, disaster risk reduction, EESI, Environmental and Energy Study Institute, ENVI-met, software to simulate climates in urban environments and assess the effects of atmosphere, vegetation, architecture and materials, FEFLOW, Finite Element subsurface FLOW system, Flood Modeller, simulates the flow of water through river channels, urban drainage networks and across floodplains using a range of 1D and 2D hydraulic solvers., FUNWAVE-TVD, fully nonlinear Boussinesq wave model, GIFMOD, Green Infrastructure Flexible Model, GSFLOW, Coupled Groundwater and Surface-Water Flow, HBV, Hydrologiska byråns vattenbalansavdelning, HEC-GeoRAS, Hydrologic Engineering Center Geospatial River Analysis System, HEC-HMS, Hydrologic Engineering Center Hydrologic Modelling System, HEC-RAS, Hydrologic Engineering Center River Analysis System, HMH, hydro-meteorological hazards, HMR, hydro-meteorological risks, HSPF, Hydrological Simulation Program - FORTRAN, HYDROBAL, eco-hydrological modelling approach for assessing water balances, HYDRUS, hydrological modelling. License Public domain software, ISBA, Interaction Soil Biosphere Atmosphere, LISFLOOD-FP, Two-Dimensional Hydrodynamic Model specifically designed to simulate floodplain inundation, MCDA/MCDM, multicriteria decision analysis/making, MIKE-SHE, integrated hydrological modelling system for building and simulating surface water flow and groundwater flow, MODFLOW, Modular Finite-difference Flow model, NBS, nature-based solutions, NHWAVE, Non-Hydrostatic Wave Model, NPV, Net Present Values, ParFlow-TREES, Terrestrial Regional Ecosystem Exchange Simulator, PLAXIS, Geotechnical Finite Element Analysis Software, QGIS, Quantum Geographic Information System, RBM, Root Bundle Model, RCM, non-hydrostatic regional climate model, RH, relative humidity, SDM, System Dynamics Modelling, SHETRAN, Distributed River Basin Flow and Transport Modelling System, SI, Supplementary Information, SIMGRO, SIMulation of GROundwater and surface water levels, SLR, systematic literature review, SLUCM, Single-Layer Urban Canopy Model, SSHV-2D, Integrated Two-Dimensional Slope Stability Model, SUDS, Sustainable Urban Drainage Systems, SURFEX, Surface Externalisée, SWAN, Simulating WAves Nearshore, SWAT, Soil and Water Assessment Tool, SWC, soil and water conservation, SWINGO-VFSMOD, Shallow Water table INfiltration alGOrithm Vegetative Filter Strip Modelling System, SWMM, Storm Water Management Model, SWMM-LID-GW, Storm Water Management Model Low Impact Development Groundwater, TEB, Town Energy Balance, TELEMAC, Finite Element Computer Programme, TOPMODEL, Topographic Model, tRIBS-VEGGIE, Triangulated Irregular Networks-based Real-time Integrated Basin Simulator and Vegetation Generator for Interactive Evolution, TUFLOW, Two-Dimensional Unsteady Flow, UCM, urban canopy layer model, UHI, urban heat island, UrbanBEATS, Urban Biophysical Environments and Technologies Simulator, US EPA, United States Environmental Protection Agency, VELMA, Visualizing Ecosystem Land Management Assessments, WRF, Weather Research and Forecasting, WSFS, Watershed Simulation and Forecasting System, XBeach, Nearshore Processes, Numerical models, Climate-impact mitigation, Performance evaluation, Cost-effectiveness, NBS upscaling, Nature-inspired solutions

## Abstract

Nature-based solutions (NBS) for hydro-meteorological risks (HMRs) reduction and management are becoming increasingly popular, but challenges such as the lack of well-recognised standard methodologies to evaluate their performance and upscale their implementation remain. We systematically evaluate the current state-of-the art on the models and tools that are utilised for the optimum allocation, design and efficiency evaluation of NBS for five HMRs (flooding, droughts, heatwaves, landslides, and storm surges and coastal erosion). We found that methods to assess the complex issue of NBS efficiency and cost-benefits analysis are still in the development stage and they have only been implemented through the methodologies developed for other purposes such as fluid dynamics models in micro and catchment scale contexts. Of the reviewed numerical models and tools MIKE-SHE, SWMM (for floods), ParFlow-TREES, ACRU, SIMGRO (for droughts), WRF, ENVI-met (for heatwaves), FUNWAVE-TVD, BROOK90 (for landslides), TELEMAC and ADCIRC (for storm surges) are more flexible to evaluate the performance and effectiveness of specific NBS such as wetlands, ponds, trees, parks, grass, green roof/walls, tree roots, vegetations, coral reefs, mangroves, sea grasses, oyster reefs, sea salt marshes, sandy beaches and dunes. We conclude that the models and tools that are capable of assessing the multiple benefits, particularly the performance and cost-effectiveness of NBS for HMR reduction and management are not readily available. Thus, our synthesis of modelling methods can facilitate their selection that can maximise opportunities and refute the current political hesitation of NBS deployment compared with grey solutions for HMR management but also for the provision of a wide range of social and economic co-benefits. However, there is still a need for bespoke modelling tools that can holistically assess the various components of NBS from an HMR reduction and management perspective. Such tools can facilitate impact assessment modelling under different NBS scenarios to build a solid evidence base for upscaling and replicating the implementation of NBS.

## Highlights

• There are no holistic models that integrate the functions, benefits and costs of NBS.
• Numerical models with some improvements are promising for NBS efficiency evaluation.
• Data intense and computational difficulties are the key cons of numerical models.
• Cost–benefit/multicriteria decision analysis methods are used to monetise NBS projects.
• The existing numerical models are lacking modules to optimise different NBS measures.

## Graphical abstract

**Figure:**
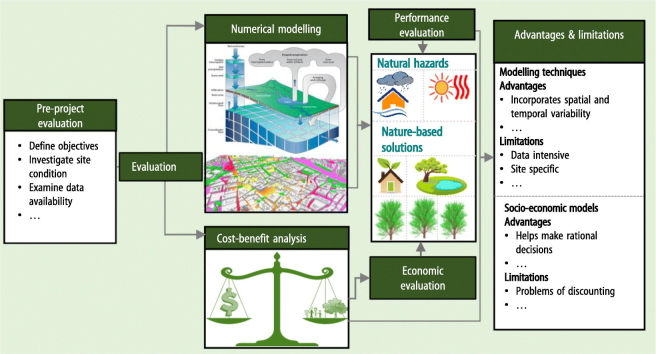

## 1 Introduction

Nature-based solutions (NBS) are interventions inspired and supported by nature which aim to ameliorate societal challenges in a cost-effective manner, while providing human well-being and biodiversity benefits (e.g., European Commission, 2016; Debele et al., 2019). In recent years, NBS have received momentum due to their multifunctional ability to counteract hydro-meteorological hazards (HMHs) and to provide multiple additional (co)benefits to human communities. However, NBS could be unable to provide ecosystem services and co-benefits until they effectively manage and mitigate the hazards under concern. HMHs, such as floods, droughts, heatwaves, landslides, and storm surges and coastal erosion, are natural phenomena that induce fatalities and economic losses in each dwelled continent (Debele et al., 2019; Paul et al., 2018). They accounted for almost 90% of major disasters around the world in the past 20 years (Wannous and Velasquez, 2017), causing long-term physical and social damage (Alcántara-Ayala, 2002). Global warming and the ensuing intensification of the water cycle have been associated with the increase in frequency and magnitude of extreme hydro-meteorological events (IPCC, 2014; Forzieri et al., 2016). There is evidence suggesting that NBS can effectively contribute to regulate the bio-geophysical processes driving HMHs (Nelson et al., 2020), while delivering co-benefits which artificially constructed concrete or grey infrastructure cannot provide (Anderson and Renaud, 2021). Examples of these co-benefits are the provision of natural capital, green jobs, clean air, water regulation, access to green spaces, recreational opportunities, or urban regeneration (e.g., Raymond et al., 2017a). However, the general uptake of NBS is still slow due to the lack of internationally recognised and comparable standard methods for assessing their multi-functional performance, hindering the establishment of a solid evidence base showcasing the benefits of NBS over conventional grey approaches for hydro-meteorological risks (HMRs) management (e.g., Nelson et al., 2020).

**Fig. 1:**
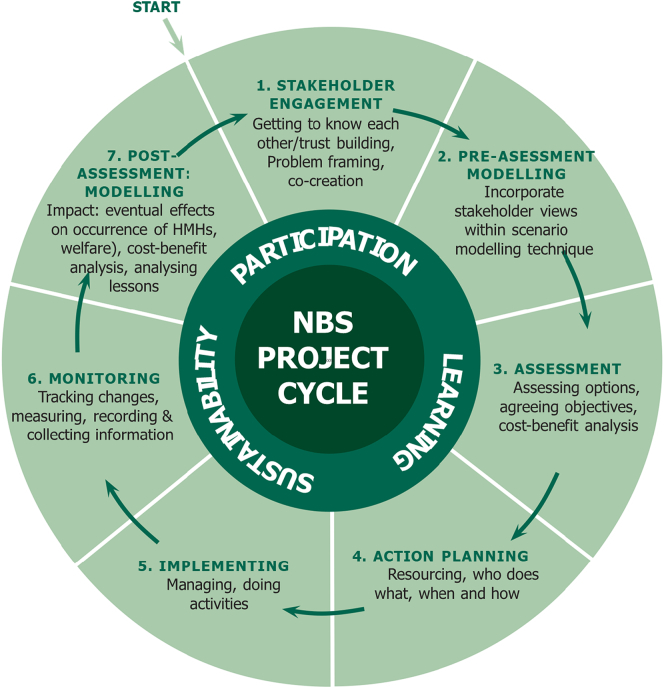
Process for executing NBS projects: the focus of this review lies on efficiency evaluation modelling (pre-and post-assessment, Steps 2 and 7) based on produced cost-benefits of the NBS interventions throughout its life cycle.

The effective NBS performance assessment requires a range of methods, models and tools aligned with all phases of NBS-project life cycle (Fig. 1). It incorporates all the activities done pre- and post-NBS implementation to establish the project objectives, understand local conditions, design the NBS intervention and choose the appropriate assessment approach for performance, sustainability and cost-effectiveness (Schwilch et al., 2011; Gachango et al., 2015; Kumar et al., 2020). NBS implementation comprises three key processes: (i) co-planning, (ii) co-design, and (iii) co-management (Kumar et al., 2020). The bio-geophysical modelling can be done during co-planning and co-management phases of NBS project life cycle. Pre-assessment modelling (Fig. 1, Step: 2) helps evaluate the selected/surveyed NBS alternatives and include the stakeholders' view before the NBS implementation as a component of the scenario modelling during co-planning phase, i.e., feasibility study. Post-assessment modelling (Fig. 1, Step: 7) is carried out to evaluate the benefits and impact of the implemented NBS project with different time horizons during co-management phase for upscaling and replicating the NBS measure to the other places. These modelling are key to evaluate the success, costs and benefits at halfway, throughout the project, and even after the project closure. For example, project evaluation using numerical modelling is done before project implementation (ex-ante) and after project closure (ex-post). Evaluating temporal changes in society and environment induced by NBS is essential for demonstrating its performance. This can encourage citizens' involvement and create trust among stakeholders during the NBS implementation phase and beyond (Kabisch et al., 2017a; Kumar et al., 2020). However, NBS assessment is complex and involves a combination of an eclectic array of quantitative and qualitative variables, some of which are hard to measure by monitoring (e.g., Kumar et al., 2021) or subjected to high degrees of uncertainty. Consequently, the use of models can help to deal with such complexity by allowing testing multiple and future socio-ecological scenarios along the projects' life cycle, providing fresh intelligence, facilitating the communication process between stakeholders and thus informing the decision-making process (Gonzalez-Ollauri et al., 2020). The use of models can also establish a good basis for merging numerical models assessing how NBS deals with HMHs with other systematic approaches evaluating the provision of ecosystem services and co-benefits, such as cost-benefit analysis (CBA). Hence, modelling can help evaluate the multi-functional performance of NBS (i.e., environmental, social, and economic), thus contributing to generate a strong evidence base on NBS performance. The challenge is to develop a balanced combination of experiment and modelling. Field experiments on NBS interventions provide evidence on the bio-geophysical performance of a specific NBS intervention. Numerical experiments yield estimates of the performance of a system of NBS interventions and address the probabilistic dimension of HMR assessments.

Numerous review articles have covered several approaches for the assessment of HMH and their management strategies. A majority of these articles focused on the monitoring methods for NBS assessment (Kumar et al., 2021), HMRs mapping and damage assessment (Teng et al., 2017; Khan et al., 2020; Sahani et al., 2019), life cycle appraisals including the economic valuation of ecosystem services (Newman et al., 2017; Eckhardt et al., 2019; Ovando and Brouwer, 2019; Nguyen et al., 2020), assessment frameworks for NBS (Dumitru et al., 2020; Shah et al., 2020), upscaling and replication of NBS (Saleh and Weinstein, 2016), and real-time forecasting of HMHs and/or HMRs. Zhang and Chui (2019) reviewed and presented models to evaluate the performance of green infrastructure in reducing runoff. They assessed the strategies for optimally allocating and designing NBS in shallow groundwater areas and highlighted that numerical modelling, and in-situ and laboratory monitoring methods can be applied simultaneously as engineering guidance and robust evaluation framework to understand the performance of green infrastructure. Ruangpan et al. (2020) presented an overview on the NBS interventions' scales (i.e., small and large), examined the existing methods for NBS appraisal and outlined the major socio-economic factors affecting the implementation process of NBS. Supplementary Information (SI) Table S1 presents a comprehensive summary of the relevant review articles on the assessment of natural hazards, their management strategies and efficiency evaluation methods. These studies usually covered only one type of HMHs along with a few elements of NBS. None of them has explicitly and extensively focused on cutting-edge modelling methods for evaluating the performance of NBS for different types of HMHs and their associated risks. Thus, there is a lack of information regarding a holistic and integrated set of modelling tools that are able to support the design and evaluate the performance of NBS for its multi-functions and benefits to the community and environment under current and/or future climate and land use.

The aim of this study is to bolster the evidence base on NBS performance by reviewing the state-of-the-art on modelling tools for evaluating the efficiency of NBS against HMHs and the associated provision of co-benefits and ecosystem services. Herein, we focus on the five types of HMHs with the most severe impacts on human life and property worldwide (i.e. floods, droughts, heatwaves, landslides, storm surges and coastal erosion; Debele et al., 2019) and we set the following four objectives: (i) systematically identify and compile the numerical models used for the optimum allocation, design, and performance evaluation of NBS; (ii) highlight the advantages and limitations of the reviewed numerical models; (iii) discuss cost-benefit analysis approaches for the cost-effectiveness appraisal of NBS projects along with their pros and cons; and (iv) recommend future course of action to further improve the NBS evidence base.

## 2 Methods, scope and outline

We adopted the systematic literature review (SLR) approach for identifying, screening, and filtering suitable peer-reviewed and grey (not published in academic journals) literature from different scientific databases (i.e., Web of Science, Scopus, ScienceDirect and Google Scholar). These are exhaustive databases, encompassing a wide span of subjects. SI Fig. S1 presents the steps adopted in this review work, indicating the number of articles identified by our searches and included/excluded for peer-review. Some pertinent papers might have been missed from our review due to the reasons as follows: (i) we restricted our review to articles published in English language and issued between 1978 and 2021; (ii) we used a specific set of keywords for the database search.

The scope of this review is limited to the application of modelling tools for the evaluation of NBS performances against floods, droughts, heatwaves, landslides, and storm surges and coastal erosion. We selected these five HMHs for analysis as they have the most severe impacts in terms of casualties, property damage and economic loss in Europe and elsewhere with significant regularity and/or intensity. For example, these five HMHs accounted for about 80.6% loss of life, and 75.2% economic losses in Europe while the remaining fractions were contributed by the other hazards (e.g., earthquake, forest fires, volcano, etc.). The corresponding contributions by these five hazards across the world were about 43.5% loss of life and 74.5% economic damages (Debele et al., 2019; Kumar et al., 2020). For NBS evaluation methods, we focused on reviewing (i) numerical or process-based models, and (ii) CBA and multi-criteria decision analysis/making (MCDA/MCDM). HMR mapping and physical damage evaluation are beyond the scope of our review paper. NBS is flexible and considered as a no-regret measure; it provides a wide range of direct benefits and co-benefits that go beyond the function of buffering HMHs at different scales. These co-benefits are not discussed in detail and rather the readers are directed to the relevant literature, keeping the main scope of the paper to the evaluation of NBS effectiveness for HMR reduction and associated monetary benefits.

**Fig. 2:**
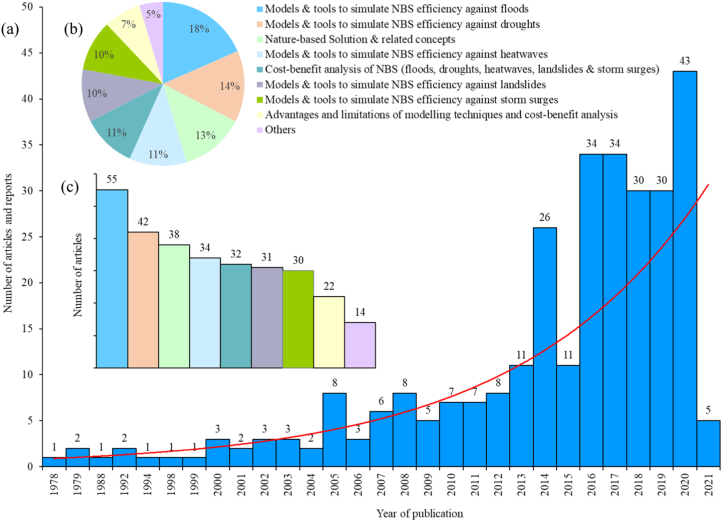
Full-text articles (298) considered in this review: (a) number of publications by year and (b) percentage and (c) number of publications by topic.

SI Table S2 lists the keywords used for the database search. This search resulted in over 7873 publications including reports, reviews, and research papers. An initial screening discarded 7575 publications whose titles and/or abstracts did not fit the scope and goal of this review. The remaining 298 articles were found suitable for full text review. Fig. 2a depicts the distribution of the selected articles per year of publication and topic, which reveals an exponential increase in NBS related studies after 2010. Among these, 64% dealt with the models and tools to simulate NBS efficiency against HMHs (floods, 18%; droughts, 14%; heatwaves, 11%; landslides, 10%; and storm surge, 10%) and the remaining 36% covered NBS (13%), CBA of NBS (11%), advantages and limitations of modelling techniques and CBA (7%), and other aspects (5%) (Fig. 2b and c).

This article is organised into seven sections: Section 1 covers the background and importance of efficiency modelling of NBS for HMH, past works on the topic and the need for this review. Section 2 explains the adopted review methodology. Section 3 discusses different NBS modelling and evaluation approaches along with their required input parameters and indicators. Section 4 analyses the advantages and disadvantages of these modelling techniques. Section 5 extends the NBS efficiency modelling discussion by including cost and benefit factors of their socio-economic impacts, rather than just biophysical considerations for their selection. Section 6 underscores the research gaps and potential way forward for further research, considering current challenges in developing an NBS modelling framework. Section 7 presents the conclusions.

## 3 Assessment framework: overview of modelling approaches

**Fig. 3:**
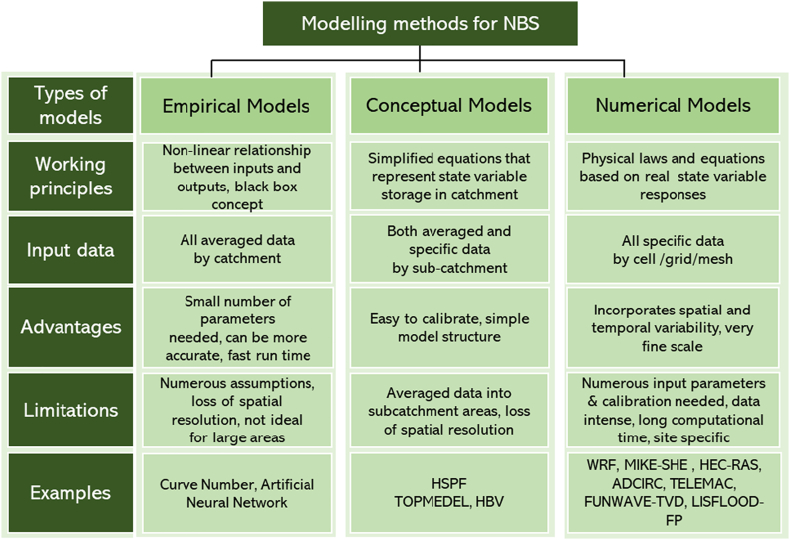
Different model structures along with the working principles, advantages and limitations, input data, and examples of different models.

Fig. 3 summarises numerous modelling methods. These differ in accuracy and complexity but could help strategic planning and designing of NBS for HMH reduction and management (Deak-Sjöman and Sang, 2015). These methods have drawn attention towards simulating the efficiency of NBS against HMH and have been included in a number of NBS projects in close collaboration with stakeholders from different sectors. Depending on their use and mathematical formulations, the model structure can be empirical (e.g., Artificial Neural Networks; Schumann et al., 2009; Devia et al., 2015), conceptual (e.g., HBV, TOPMODEL, HSPF; Devia et al., 2015; Johnson et al., 2003), and process-based or numerical (e.g., MIKE-SHE, WRF, SWMM; Devia et al., 2015; Brunner, 2016; Moulinec et al., 2011; Vacondio et al., 2011). Empirical models are widely intuitive but are only reliable when applied to scenarios similar to those used as a reference for their build up. Numerical models tend to be more sophisticated and computationally demanding. They solve the mathematical equations describing the physical phenomena under simulation (e.g., conservation of momentum, mass, and energy for simulating water and air flow). This physically-based simulation allows assessing new NBS scenarios. Zhang and Chui (2019) concluded that the simulations of process-based models tend to be more effective and provide more robust results for NBS design and their in-situ monitoring than empirical and conceptual models. Based on their spatial characterisation, these can be categorised into one dimensional (1D), two dimensional (2D) or three dimensional (3D) models. The structure of the model determines how input data is used to map HMR, assess the damage and simulate NBS remediation effects. However, different modelling approaches provide different capabilities for evaluating the NBS efficiency.

Numerical models can be grouped into three types (e.g., Zhang and Chui, 2019): green approach-explicit surface-subsurface models (e.g., ENVI-Met, SWMM-LID-GW, SWINGO-VFSMOD, GIFMOD), surface-subsurface hydrologic models (e.g., MODFLOW, SWAT, MIKE-SHE, VELMA, SHETRAN, GSFLOW) and variably saturated permeable media models (e.g., FEFLOW). Zhang and Chui (2019) also categorised numerical models for performance evaluation of NBS at planning (e.g., multiple green infrastructure practices at watershed scale) and design levels (e.g., individual green infrastructure) based on their capabilities and potential applications. For instance, some studies have applied numerical models such as hydrodynamic models (e.g., MIKE-SHE, SWAT, MODFLOW) for evaluating the hydrological efficiency of green infrastructure (Zhang and Chui, 2017, Göbel et al., 2004; Newcomer et al., 2014; Thompson et al., 2010; Stewart et al., 2017; Joyce et al., 2017). 1D, 2D and 3D hydrodynamic models (e.g., SWMM, UrbanBEATS, MIKE-SHE, HEC-RAS, HEC-GeoRAS, SWAT, Flood Modeller, LISFLOOD-FP, ADCIRC, TELEMAC) have been utilised to evaluate the performance of NBS (e.g., wetland, ponds, bio-retention, grass swale, porous pavement, salt marshes, sea grass) against floods, storm surges and droughts (Tayefi et al., 2007; Guida et al., 2015; Niu et al., 2016; Yeo et al., 2019; Jamali et al., 2018; Highfield et al., 2018). A few studies (e.g., Joyce et al., 2017; Bach et al., 2020) developed a multi-scale modelling technique to evaluate the efficiency of a green-NBS. In addition, the System Dynamics Modelling (SDM) approach is being increasingly utilised for the assessment of NBS effectiveness, with a particular focus on their ability to provide multiple co-benefits, such as nature conservation, human health, and well-being, besides buffering communities from HMRs. The application of SDM techniques could support studying the behaviour of complex systems through time by changing the whole system into a set of variables that are interrelated through feedback loops (Chen and Wei, 2014; Zomorodian et al., 2018). For instance, Pagano et al. (2019) developed a participatory SDM framework for the stakeholders' evaluation of NBS multi-dimensional impacts throughout its project life cycle. Such a framework has been implemented in the Glinščica river (Ljubljana, Slovenia) to quantitatively evaluate the effectiveness of NBS to handle flood risks. The study highlighted that the applicability and effectiveness of the framework was hindered by some elements of uncertainty, such as insufficient spatial information or the lack of an economic assessment of the chosen strategies and requiring further research before its consideration to support decision-making processes. All these models and tools are utilised to: (i) understand the driving processes of HMHs in hazard-prone zones; (ii) predict the occurrences of HMH using proxies or indicators (e.g., water level, temperature), and (iii) simulate NBS performance against HMHs and enable adaptive management of the NBS (e.g., which plant cover is more effective against landslides?; which minimum water level is sustainable against drought?). The advantages of using numerical models to achieve the former goals are: (i) handling, merging and simplifying realistically complex environmental scenarios and processes; (ii) undertaking long-term predictions beyond the project's timescale and detecting emergent properties of the ecosystems under study, (iii) managing the sensitivity and uncertainty associated with the environmental processes modelled and their input variables, and (iv) setting and assessing multiple case scenarios of climate, land cover, socio-economic contexts, and/or NBS management. In general, the NBS can be included into each modelling technique reviewed below (3.1, 3.2, 3.3, 3.4, 3.5) by considering land cover changes and/or through the solution of boundary value problems, which are generally input into the models as geospatial datasets (e.g., shapefiles). Yet, the underlying mechanisms by which NBS regulate the drivers triggering HMHs must be incorporated in the models through the modification of key, sensitive variables/proxies/indicators or through changes in the mathematical functions explaining their behaviour numerically. Given that there is a severe lack of evidence base of how NBS perform (Ruangpan et al., 2020), the use of models opens an exciting opportunity to foresee NBS performance and contributes to overcome the knowledge gap obstacle for their implementation.

### 3.1 Flood

The modelling techniques for flood inundation and flood frequency analysis have significantly improved in the last half century (Teng et al., 2017; Debele et al., 2017a; Debele et al., 2017b; Debele et al., 2017c). The models incorporating NBS for flood remediation have broadly been used in flood risk assessment and mapping (Li et al., 2019a, Li et al., 2019b; Thorslund et al., 2017; Wu et al., 2019; Martinez-Martinez et al., 2014; Yeo et al., 2019; Vinten et al., 2019; Guida et al., 2015; Vuik et al., 2018; Jurczak et al., 2018; Alves et al., 2020; Lin et al., 2020), flood damage assessment (Ming et al., 2007; Alves et al., 2020) and coastal flood risk mapping (Vuik et al., 2016; Highfield et al., 2018; Wamsley et al., 2010; Liu et al., 2019; Stark et al., 2016). Here, we briefly discuss the models which have been used to simulate the efficiency and performance of NBS, such as wetlands, ponds, and green approaches against flood risk along with their scale and input data.

Table 1 presents a comprehensive summary of models used to simulate the efficiency and performance of NBS against flood risk. The Hydraulic Engineering Centre-River Analysis System (HEC-RAS) is a widely used hydraulic model for determining inundation extent, mapping the flood risk and simulating the effect of NBS designs (Guida et al., 2015; Tayefi et al., 2007). These models can simulate scenarios at high spatial and temporal resolution (usually ranging from 5 m to 2000 m and less than 0.1 s to 24 h). HEC-RAS models require the following input data: (i) geometry data (shape, size, elevation and connectivity of stream cross-sections), (ii) boundary and initial conditions data (flow or water depth), and (iii) geospatial data, which can also be included to overlay the model on georeferenced maps (Psomiadis et al., 2021; HEC-RAS, 2016). For example, Guida et al. (2015) used 1D and 2D HEC-RAS hydraulic models and investigated the floodplain reconnection and the role of wetlands in attenuating flood waves using hydrologic and geospatial data for the Lower Tisza District in Hungary. These data included daily river level measurements, observed daily river discharges, a 5 m digital elevation model (DEM), shapefiles including river levees location and population data. Elevation data were extracted from DEM cross-sections, while wetland areas were identified and digitised from georeferenced historic maps. Thomas and Nisbet (2007) used HEC-RAS to simulate the effect of woodland on changes in peak flow, velocity and stage, travel time and storage volume at a 2.2 km reach in River Cary, UK. The surface runoff was simulated for three scenarios: the existing situation, a complete woodland cover and a partial woodland cover. They found that woodland delayed the flood peak arrival and lowered the peak discharge but increased the duration of the flood event. Tayefi et al. (2007) applied a novel framework of hydraulic (1D and 2D HEC-RAS) and geospatial modelling (HEC-GeoRAS) to determine the optimal flood risk reduction measures. They simulated two scenarios, considering the presence of a levee and its removal to reconnect the river and wetland, and found that the wetland connection significantly reduced flood depth and potential damage to human populations.

**Table 1:** Overview of input hydroclimatic variables used to understand flood risk and numerical models used to simulate the efficiency and performance of NBS against flood risk.

Purpose	Type of NBS (place)	Models to simulate NBS efficiency	Input hydroclimatic parameters	References
To study the potentials of wetlands using the SWAT module of a GIS platform.	Wetlands (Bojiang Haizi River, Erdos *Larus relictus*)	SWAT	Daily rainfall, wind speed, RH, solar energy and air temperature	Li et al. (2019a)
To study the effects of vegetation on flood wave attenuation on the basis of a combination of field observation and numerical modelling.	Salt marshes and coastal wetlands (Western Scheldt estuary, the Netherlands)	SWAN numerical wave model	Field measurement, bathymetry, ocean current, ocean water level, bottom fraction, and wind speed.	Vuik et al. (2016)
To assess the functions of estuarial and tidal wetlands in reducing storm surge and flood damages.	Estuarine wetlands (mudflats and channels) (USA)	ArcGIS, ADCIRC numerical model	Wind velocity and atmospheric pressure	Highfield et al. (2018)
To simulate the role of wetland and vegetation roughness in reducing storm surge effects.	Wetland and vegetation roughness (Southeast Louisiana)	ADCIRC simulation/regression analysis	Wind velocity, atmospheric pressure, topo bathymetric, manning coefficient	Barbier et al. (2013)
To develop methods to delineate wetland inundation extent at basins.	Wetlands (Prairie Pothole, central North Dakota)	LiDAR, ArcGIS	Multi-temporal NAIP imagery, national wetlands inventory dataset, NDVI	Wu et al. (2019)
To study the effects of wetland regions their depth and positioning on river flows and peak flow control at basin scale.	Wetlands/ponds (Shiawassee River watershed, Saginaw Bay)	SWAT	Land use, soils, wetland field data, precipitation, RH, potential evapotranspiration	Martinez-Martinez et al. (2014)
To simulate hydrological processes with and without geographically isolated wetlands.	Contracted wetland (Greensboro Watershed, Mid- Atlantic Region of USA)	SWAT-WET	DEM, wetland drainage zones, daily precipitation temperature, and streamflow.	Yeo et al. (2019)
To analyse the role of weir and dredging of the channel in reducing upstream flood risks.	Wetland conservation, pond, lake (upper Lunan basin Scotland)	HEC-RAS	Maximum elevation, river water levels, discharge, lake water levels, precipitation	Vinten et al. (2019)
To simulate the potential of wetlands in attenuating peak water levels during storm tides.	Hybrid (Western Scheldt estuary, the Netherlands)	2D hydrodynamic model (TELEMAC 2D)	DEM, hourly averaged wind speeds, water level.	Stark et al. (2016)
To apply a novel framework of hydrodynamic and geospatial modelling to simulate the optimal flood risk reduction measures by wetland.	Wetland (Lower Tisza River, Hungary)	1D HEC-RAS model, ArcGIS, HEC-GeoRAS	DEM, daily discharge, maximum annual discharges, levees height	Guida et al. (2015)
To present a method that can describe the failure likelihood of a hybrid flood water protection system by integrating numerical models with stochastic models.	Hybrid flood (Netherlands)	1D wave energy balance	Mean wave period, water level, significant wave height, and wind speed	Vuik et al. (2018)
Using the hybrid (blue-green) approach to retain and purify stormwater runoff from the street.	Hybrid (blue green) (Łódź, Poland)	Field survey	Precipitation, discharge	Jurczak et al. (2018)
Effectiveness of several NBS in the reduction of runoff.	Bio-retention, grass swale, and porous pavement (Tianjin University, China)	Storm Water Management Model (SWMM)	Precipitation, Temperature, Evaporation, Wind speed, Basin elevation	Niu et al. (2016)
Investigating whether an increase in the number of nature-based features can reduce surface runoff in hillslope areas.	Low earth bunds and debris dams (Brompton catchment, UK)	TOPMODEL and 1-dimensional hydraulic channel routing scheme	Precipitation, digital elevation model	Metcalfe et al. (2017)
Effect of applying NBS on several hydrological variables related to floods.	Tree woodland (River Cary, UK)	HEC-RAS and 2-dimensional River2D hydraulic model	Precipitation, River channel, river cross section	Thomas and Nisbet (2007)
Simulating changes in flow of water along channels and across surfaces due to application of NBS.	Storage pond (Tarland Burn catchment, UK; Spercheios River Basin, Greece)	TUFLOW	Precipitation, Basin boundary, Initial water level, Land use, Soil infiltration, Elevation	Ghimire et al. (2014) Spyrou et al. (2021)
Potential of green infrastructure in regulating surface runoff under climate change scenarios.	Trees and green roofs (Munich, Germany)	MIKE-SHE	Precipitation, Basin boundary, Manning's number, Wind speed, Evaporation, Temperature	Zölch et al. (2017)
Reduction of flood damages during coastal flooding	Coastal wetlands (New Jersey, USA)	MIKE-21	Precipitation, Basin boundary, Manning's number, Wind speed, Evaporation, Temperature	Narayan et al. (2017)
Investigating the synergic effects of floodplain restoration on flood risk reduction	Forest and wetland revegetation (Vermont, USA)	HEC-RAS and economic flood damage cost model	Precipitation, River channel, river cross section	Gourevitch et al. (2020)
A hydrodynamic approach is combined with an optimisation function to assess various green, blue and grey solutions in an integrated way.	Green-blue-grey approach (Sint Maarten Island, Saint Martin)	Hydrodynamic model EPA SWMM coupled with optimisation algorithm, Questionnaire, multi-criteria analysis	Model simulated precipitation data and evaporation	Alves et al. (2020)
To evaluate the efficiency of isolated wetland subsurface and surface hydrologic connections to rivers.	Wetland soils (Prairie Pothole, North America) (Prairie Pothole Region of North America)	HydroGeoSphere model	DEM, water level, rainfall	Ameli and Creed (2017)
To evaluate the performance of dune structure reconstruction as a DRR solution in the face of current and future sea level conditions at a quickly eroding coastal area.	Dune system rehabilitation (reconstruction and revegetation), Bellocchio, Italy	Hydro-morpho dynamic model	Temporal analogue extreme storm event from 5 to 6 February 2015, used to test the NBS	Fernández-Montblanc et al. (2020)
A societal scale model was built to estimate the efficiency of green NBS on reducing the magnitude and quick flow of urban surface runoff.	Green infrastructure, Beijing, China	Community scale simulation model	Urban flooding	Liu et al. (2014)
To estimate overall benefits of flood storage capacity which was implemented as part of the restoration of wetlands in this area.	Wetland and ponds (Cambridgeshire, UK).	TESSA toolkit		Peh et al. (2013)
To estimate the impact of shore area wetlands in the northeastern USA against hurricane induced flood risk.	Coastal wetland cover (Atlantic coast USA),	MIKE-21 flood model	The model was simulated by the wind which was based on observed data Bathymetry data was part of the MIKE model C-MAP.	Narayan et al. (2017)
To offer a worldwide study of the socio-economic value of mangroves for flood risk management.	Mangrove forests, global analysis.	Delft3D	Historical cyclones and normal waves and sea level astronomical, storm surge, tide and mean sea level to generate the regression model	Menendez et al. (2020)
To present a methodology for the choice and placing of NBS to accomplish urban flood risk management.	Green wall/roofs, bio-retentions, rain gardens and previous pavements, Sukhumvit area, Bangkok, Thailand	A macro scale approach for urban flood modelling, using the Mike Urban hydrodynamic model.	Rainfall return periods (1-in-2 year, and 1-in-20 year)	Majidi et al. (2019)

MIKE-SHE is a 1D, 2D and 3D hydrological and hydraulic modelling system capable of simulating overland and soil flow, as well as water quality processes in rivers, floodplains, wetlands and reservoirs. Many studies have applied 1D MIKE 11 coupled with MIKE-SHE (e.g., Thompson et al., 2004; Clilverd et al., 2016) or alone (e.g., Thompson et al., 2017; Clilverd et al., 2016) to simulate the potential of NBS for mitigating flood risks and climate change impacts in many parts of the world. The basic dataset required to simulate, for instance, the effect of floodplain restoration is: (i) pre- and post-restoration topography, (ii) discharge and meteorological data (daily precipitation, potential evapotranspiration and observed groundwater elevations), and (iii) hydraulic geometry. The temporal and spatial discretisation of the model ranges from 1 to 30 min and 1–1000 m, respectively. Zölch et al. (2017) assessed the potential of green NBS (green roofs and trees) in controlling urban flooding in the current and projected (2030–2060) climate scenarios for storm events of different return periods or probability of occurrence in Munich, Germany using the MIKE-SHE model. They found a maximum reduction in peak flows of 14.8%, which was highly associated with shares of green cover compared to the baseline scenario. Metcalfe et al. (2017) evaluated nature-based in-channel features/barriers, such as low earth bunds and debris dams to create storage, increase the subsurface flow and reduce the surface runoff or peak discharge during a storm event in a hillslope area at the Brompton catchment, UK, using a coupled hydrological-hydraulic model. TOPMODEL (semi-distributed hydrological) model was used to mimic hillslope overflow into the river channel, and a 1D hydraulic channel routing scheme was used to model the water levels, flow velocities in the river network and to connect the river channel with the floodplain. The study found that the optimal number of barriers in the area was 59, which can reduce the peak discharge by 10.6% and a delay in peak by 2 h 45 min. Other hydrodynamic models such as MODFLOW, HYDROBAL, SWMM and HYDRUS models are also used to simulate the efficiency of different NBS designed to reduce flood risks.

Storm Water Management Model (SWMM) developed by the US EPA is an integrated hydraulic and hydrological modelling tool which is broadly used to assess the efficiency of low-impact development measures in urban environments (Zhang and Chui, 2018). SWMM is widely used for the analysis and design of urban drainage systems (e.g., Elliott and Trowsdale, 2007; Jayasooriya and Ng, 2014; Zhang and Chui, 2020). It simulates the rainfall-runoff transformation using a catchment-based lumped approach and the conveyance of stormwater, sewage and pollutants in the drainage network using hydraulics numerical methods. SWMM can also simulate losses due to infiltration and evaporation, and runoff retention and ponding. It can be used to evaluate grey approaches to rainwater drainage (e.g., storm drains and pipes) and is an effective model for establishing cost-effective hybrid (e.g., green-grey) NBS as rainwater control measures. For instance, it can explicitly simulate the effectiveness of hybrid NBS, such as rain gardens, continuous permeable pavement systems, rainwater harvesting, green roofs, rooftop, vegetative swales, bioretention cells/bioswales and infiltration trenches against flooding at the urban scale (Nizzi et al., 2017). The input data requirements are: (i) a land surface component containing the definition of sub-catchments and their runoff characteristics; (ii) a conveyance system component, which describes pipes, channels, flow regulators, and storage units; (iii) external forcing data (precipitation, temperature, evaporation); (iii) a subsurface groundwater component; (v) contaminant build-up, wash-off, and treatment; and (vi) LID controls to represent combinations of green-grey infrastructure practices. Zhang and Chui (2020) integrated the modified SWMM which is called SWMM-LID-GW, with MODFLOW to form a loosely-coupled surface-subsurface hydrological model (SWMM-MODFLOW) that can evaluate the surface runoff and groundwater table dynamics of NBS (e.g., bioretention cells) of various spatial apportionments at a watershed scale. Based on the simulation results, they concluded that the effectiveness of spatial apportionments of NBS (e.g., bioretention cells) depends on (i) the aggregation level, (ii) the execution ratio, and (iii) the relative location of bioretention cells in the catchment. Zhang et al. (2018) also used SWMM-LID-GW to simulate water movement in the soil and its interaction with the surface for a more holistic performance assessment of NBS. Niu et al. (2016) analysed the effect of bio-retention, grass swale and porous pavement in the reduction of runoff at the Tianjin University campus (2.5 km^2^), China, using the SWMM software. Based on the 2D grid-based hydrodynamic model called TUFLOW, Ghimire et al. (2014) simulated the water flow along channels and across surfaces at the Tarland Burn sub-catchment (74 km^2^) of the River Dee. They showed that a pond as an NBS reduced the peak discharge and that this reduction was positively related to the storage capacity.

Flood Modeller (1D and 2D) is a hydrodynamic model developed to solve the shallow water equations (Jamali et al., 2018). Flood Modeller simulations require the following input datasets: river networks, event data (rainfall events, historical river discharge, water level), boundary conditions (1D and 2D), cross sections (for 1D). In the simulation, flood risk NBS intervention can be introduced in the form of weirs which will create mill ponds and help to attenuate flood peaks and prevent the associated risks. LISFLOOD-FP is another hydrodynamic modelling tool able to solve the 1D open channel shallow water equations which takes advantage of DEM for flood mapping (Neal et al., 2018; Sosa et al., 2020). The model has been successfully used to simulate NBS performance with DEM grid resolutions of 25–100 m and time steps ranging from 2 to 20 s. The input data requirements are: (i) raster DEM, (ii) boundary conditions in the form of hydrographs or time-varying water surface elevation, (iii) rainfall, (iv) channel geometry, (v) channel and floodplain friction. Other hydrodynamic models, such as HydroGeoSphere (e.g., Ameli and Creed, 2017), and HEC-HMS (e.g., Tang et al., 2020) are also used to simulate flood risk maps and damage assessment along with potential NBS (Table 1).

Soil and Water Assessment Tool (SWAT) is a semi-distributed, watershed or river basin scale model designed to mimic the quantity and quality of water bodies and forecast the environmental effect of land use, land management activities, and global warming. It is widely used in evaluating soil erosion prevention by soil and water conservation measures (Melaku et al., 2018), and flood risk reduction through wetlands (Yang et al., 2016; Wu et al., 2019; Yeo et al., 2019; Li et al., 2019a). It requires the following input data: (i) watershed DEM, (ii) hydrological response units, (iii) ponds/wetlands/reservoirs shapefiles, (iv) point sources, and (v) meteorological data (daily precipitation, temperature, potential evapotranspiration, relative humidity (RH), wind speed and solar radiation). SWAT has been used to simulate NBS efficiency against flood risk and damages alone (e.g., Yeo et al., 2019; Martinez-Martinez et al., 2014) or in combination with ArcGIS (e.g., Li et al., 2019a). The ArcGIS software is a comprehensive and integrated general-purpose geographical information system developed by the ‘Environmental Systems Research Institute’ for combining and analysing geospatial data (Maguire, 2008). It allows the creation of geographical features, such as water bodies or green infrastructure in the form of shapefiles that are fed into some of the above-mentioned models. It also allows mapping and analysing the modelling results.

### 3.2 Droughts

Several modelling approaches can be utilised for evaluating the current and anticipated effects of NBS across various drought conditions (Somarakis et al., 2019). Table 2 summarises the input variables used to understand drought risk, the types of NBS used for its amelioration and the most common numerical models to simulate the efficiency and performance of NBS against drought risk. For example, ParFlow-TREES is a hydrological model that amalgamates groundwater and plant hydrology and hydraulics to evaluate the diverse response of forest to drought at the watershed scale (Tai et al., 2018). It can solve variably unsaturated and saturated soil flows in 3D utilising either a terrain-following semi-structured grid or an orthogonal grid that allows fine vertical resolution in the upper soil layers, and unconfined and deep confined aquifers. ParFlow-TREES determines the changes of shallow and sub-surface flows by optimising the surface water equations coupled with the Richards equation for soil water flow using a finite-difference approximation. It simulates the water movement following the hydraulic gradient vertically in the plant, soil and environment continuum and in the transversal direction underground (Maxwell, 2013). The input variables required are: (i) leaf and branch area index; (ii) hydraulic variables (upper and lower layer soil hydraulic conductivity at saturation, manning's coefficient, initial and boundary conditions); (iii) meteorological variables (dew-point temperature, forest albedo, air temperature, atmospheric long wave radiation, wind speed and precipitation); (iv) soil data (soil water potential, upper and lower layer soil moisture content at saturation); (v) NBS characteristics (trees: types, density, trunk size, the volume of branches and leaves, height, and rooting depth). For example, Tai et al. (2018) applied the ParFlow-TREES model to simulate plant transpiration and photosynthesis, and thus estimated the vulnerability of coastal cottonwoods in southwestern Canada to sustained meteorological drought and variation in river flow using the meteorological variables (CO_2_ concentration, atmospheric pressure, photosynthetically active radiation, temperature, wind speed, precipitation, vapor pressure deficit). The model demonstrated a sustained nexus between regional subsurface flows and the ecological processes that could help reduce hydrological drought at the landscape scale and guarantee the survival of trees or forests.

**Table 2:** Overview of input hydroclimatic variables used to understand droughts risk and numerical models used to simulate the efficiency and performance of NBS against drought risk.

Purpose	Type of NBS (place)	Models and tools to simulate NBS efficiency	Input hydroclimatic parameters	References
Hydrological and economic modelling to estimate costs and benefits of ecological restoration for increasing annual streamflow	*Re*-vegetation of hillslopes and degraded land, removal of invasive plant species.	ACRU	Terrain topography, daily rainfall, temperature, soil descriptors, land use/land cover. Restoration costs (e.g., project duration, extent of target area, degradation level, type of water yield prioritised). Benefits based on water gains and average water value.	Mander et al. (2017)
Observation to alleviate hydrological drought as part of an integrated water resource management plan.	Increasing the water table in the main waterways and increasing the beds of the small waterways.	SIMGRO distributed process-based model to simulate groundwater and streamflow time series	Terrain topography, soil type, geological strata, land use and hydrological variables.	Querner and van Lanen (2001)
To simulate plant transpiration and photosynthesis and thus estimate the vulnerability of coastal cottonwoods in south western Canada to sustained meteorological drought and variation in river flow	Trees: types, density, trunk size, volume of branches and leaves, height, and rooting depth (south western Canada)	ParFlow-TREES	Meteorological variables (CO2 concentration, atmospheric pressure, photosynthetically active radiation, temperature, wind speed, precipitation, vapor pressure deficit).	Tai et al. (2018)
Hydrological modelling to estimate the impact of global warming which could change dry spell length and the effect of drought risk on main water supply sectors.	Area specific drought reduction strategies and incorporation of droughts in current area readiness exercises.	Finnish Environment Institute's Watershed Simulation and Forecasting System (WSFS) hydrological model	Rainfall, wind speed, RH, air pressure and cloudiness, daily temperature.	Veijalainen et al. (2019)
To investigate the potential of wetlands and salt marshes to reduce drought risks in the Bojiang Haizi River basin, Erdos Larus Relictus Nature Reserve plateau.	Wetlands, salt marsh and retention ponds (Global)	SWAT	Land use, topography, soils, wetland field data, precipitation, temperature, solar radiation, wind speed, RH, potential evapotranspiration.	Li et al. (2019a)
SWEMs is an important tool to forecast the effect of meteorological variables - precipitation, atmospheric CO2 concentrations and temperature on soil erosion and agricultural drought and used to assess the effects of forest, cropland and vegetation on soil erosion and drought risk.	Forest, cropland and vegetation (Global)	Soil and Water Integrated Model (SWIM)	Temperature observed soil erosion, precipitation (rainfall, rainstorms, and freeze-thaw cycles) and atmospheric CO2 concentrations.	Guo et al. (2019)
To evaluate the efficiency of plants with deep roots to seasonal drought risk or to mimic changes in rooting depth with time.	Drought tolerant, crops, root depth (Global)	HYDRUS 2D/3D	Plant root water uptake in the horizontal and vertical directions, soil hydraulic functions and root distribution with depth.	Ghazouani et al. (2019)
To investigate vegetation and hydrological responses to global warming in a forested mountainous watershed dynamic vegetation model (LPJ) coupled with a 3D hydrogeological model (MODFLOW) to estimate the effect of global warming on a small forested temperate watershed.	Forests, vegetation, herbaceous surroundings (Strengbach, Vosges, France).	Lund-Potsdam-Jena Dynamic Global Vegetation Model (LPJ), MODFLOW	Mean meteorological data (precipitation, amount of wet days, cloud cover, air temperature), vegetation and soil.	Beaulieu et al. (2016)

Agricultural Catchments Research Unit (ACRU) model is flexible, comprehensive and can mimic river discharges, evapotranspiration, and the impact of water abstractions on the aquifer at daily time steps at sub-catchment or catchment scale (Schulze, 1995). Simulated stormflow and baseflow in streams depend on the daily precipitation with respect to the dynamics of the soil moisture budget. The model input variables are (i) air temperature, (ii) daily precipitation, and (iii) land cover type and soil characteristic of the spatial unit being modelled (Rebelo et al., 2015). Mander et al. (2017) used ACRU coupling hydrological and economic models to instigate further water-related ecological and economic investments in infrastructure in South Africa. They evaluated the efficiency of NBS consisting of thicket vegetation to enhance base-flows in dry periods and to reduce flood peaks.

SIMulation of GROundwater and surface water levels (SIMGRO) is a comprehensive, distributed and transient model that mimics surface and groundwater flow in the saturated and unsaturated zone by schematising the system geography, both horizontally and vertically at subregional and regional scales (Querner and Povilaitis, 2009) and is suitable for studying droughts (Querner, 1988). The horizontal schematisation enables the input of various soils and land cover types as sub-regions to simulate spatial variations in moisture content in the unsaturated soil (Querner and van Lanen, 2001). SIMGRO requires input data such as topography, hydrogeological parameters, land cover, soil characteristics, and geological strata. SIMGRO has been applied to evaluate current and improved water management practices in arid areas (see e.g., Querner et al., 1997) and to evaluate interventions in water management to mitigate the impact of irrigation on soil and water salinity (Kupper et al., 2002). Earlier, Querner and van Lanen (2001) applied SIMGRO in the Poelsbeek and Bolscherbeek low-land watersheds in the eastern Netherlands to alleviate hydrological drought as part of a current and future holistic water resource management approach. Increasing the water amounts in the main waterways and raising the beds of the small watercourses by weirs was used as a nature-based intervention in this model which increased the groundwater level and thereby reduced groundwater drought.

A more specialised numerical hydrological model, the Watershed Simulation and Forecasting System (WSFS) was applied by the Finnish Environment Institute (Vehviläinen, 1992; Vehviläinen et al., 2005) to forecast the effect of droughts under different climate change scenarios. Results showed that severe droughts could have a substantial effect on waterways, leading to a decrease in the water supply in Finland, with negative impacts on hydropower production and agriculture.

### 3.3 Heatwaves

The effectiveness of NBS for mitigating the impacts of heatwaves or heat stress has been studied using different modelling techniques. ENVI-met, a micro-scale 3D computational fluid dynamics (CFD) model (Wang and Akbari, 2016; Chatterjee et al., 2019; Pigliautile et al., 2020) and Weather Research Forecast (WRF) coupled with the single- or multi-layer urban canopy layer model (UC) (Imran et al., 2018; Jandaghian and Akbari, 2018 etc.) are the two most common modelling tools (Table 3), followed by others such as Ecosystem Service Model (Venter et al., 2020), Open Studio and EnergyPlus (Yang et al., 2018a), Town Energy Balance (TEB) coupled with the Interaction Soil Biosphere Atmosphere (ISBA) model (Daniel et al., 2018), Surface Energy Balance (Mariani et al., 2016), SURFEX (Broadbent et al., 2018), and TUF-3D (Yang et al., 2019). ENVI-met is commonly used to simulate air-surface-plant-interactions in urban environments (Crank et al., 2018; Tiwary and Kumar, 2014). It has been utilised in simulating the effects of buildings, streets and vegetation in the microenvironment of biomes. An urban open space model was developed using ENVI-met by Zhao and Fong (2017) for evaluating the cooling potential of different landscape designs (base, green, grey, blue and hybrid) with the aim to mitigate heat island effects and to relieve heat stress for humans. They found that hybrid-NBS had further cooling benefits compared with the singular landscape designs. ENVI-met was used with sub-module BioMet for human-biometeorological simulations for a typical heatwave day in Germany where four different urban green schemes were examined for the cooling benefit of grasslands and trees (Lee et al., 2016). Trees were found to be more effective than grasslands in mitigating human heat stress. Crank et al. (2018) investigated ENVI-met's validity regarding the surface energy balance, grid sensitivity/independence, and efficacy for assessing rooftop level heat alleviation strategies. Although ENVI-met is grid dependent, the results indicated that the extent of the software's reliability on grid resolution is smaller than the extent of the simulated effects of the alleviation plan. Therefore, the effect of grid susceptibility to moderations in vertical resolution overshadowed ENVI-met-projected impressions of heat alleviation strategies on air temperature. Identified limitations subjected to further research using ENVI-met include its accuracy for atmospheric variables other than air temperature and representation of ground to roof level vertical mixing and surface energy balance in the urban environment (Crank et al., 2018).

**Table 3:** ENVI-met model applied to the micro-meteorology simulations for evaluating different NBS performance measuring the relevant performance indicators. WRF model applied for the meso to macro scale meteorology simulations for evaluating different NBS performance measuring the relevant performance indicators.

Purpose	Type of NBS	NBS performance indicator	Reference
Quantifying cooling potential of different types of NBS	Green/grey/blue and hybrid (Hong Kong)	Reduction in temperature of air (ΔTa) and physiological equivalent temperature (ΔPET)	Zhao and Fong (2017)
Testing four different urban green scenarios for cooling effect	Trees and grasslands (Germany)	Ta, PET and mean radiant temperature (Tmrt) to represent human heat stress	Lee et al. (2016)
Evaluating the best suitable strategy to ameliorate built-up micro-scale thermal scenarios.	Green infrastructure (Sri Lanka)	Temperature reduction	Herath et al. (2018)
Finding the extent of the maximum reduction in outdoor human heat stress by urban green spaces during severe summer heat	Grassland and trees (Stuttgart, Southwest Germany)	PET, Tmrt and Ta	Lee and Mayer (2018)
Studying the effects of the gaps between tree crowns for reducing heat stress during the day for pedestrians inside E-W built-up street canyons in central European situations	Urban trees (Freiburg, Southwest Germany)	PET and Tmrt	Lee et al. (2020)
Examining the implication of green infrastructure to assess the appropriate UHI management scheme	Green roof and green wall (West Bengal, India)	Temperature profile	Ziaul and Pal (2020)
Studying the effect of heat management schemes on the surface energy balance at the neighborhood scale	Green roof and additional trees (El Monte, LA, Southern California)	surface sensible heat flux (W/m2)	Taleghani et al. (2019)
Evaluating the achievement of four kinds of heat management schemes to compensate the effect of UHI episode	Green cover (Kolkata, India)	Biophysical thermal indices (human weighted Tmrt, standard effective temperature, PET, predicted mean vote) and Thermal parameters (thermal radiative power, net radiation and urban morphological parameter: sky view factor)	Chatterjee et al. (2019)
Investigating solutions to mitigate the microclimatic conditions and improve the thermal comfort of the citizens	Canopy, water stretch, urban vegetation (Mirti square, Centocelle, Rome, Italy)	Ta, Universal Thermal Climate Index	Battista et al. (2019)
Evaluating the various schemes for UHI impact management during the day in regard to thermal relief	Urban vegetation (street and roof) (Tehran)	Sky view factor, Ta and surface temperatures, Tmrt, PET and wind speed	Farhadi et al. (2019)
Evaluating two site-specific design strategies (wind-path and sky view factor) for tree planting in the built-up conditions for UHI management	Urban trees (Hong Kong)	Solar transmissivity, surface temperature, Ta reduction, Tmrt, sky-view factor	Tan et al. (2016)
Evaluating various UHI management schemes in different built-up neighbourhoods	cool roof, cool pavement, and putting urban greenery (Toronto, Canada)	outdoor Ta, surface temperature, Tmrt, and PET, thermal radiative power and net surface radiation	Wang et al. (2016)
Comparing the effect of tree size and space between trees on outdoor comfort for the common tree types and their size	Urban Trees (Montreal, Canada)	Tmrt, Ta	Wang and Akbari (2016)
Assessing xeriscaping as a sustainable heat island mitigation strategy.	Xerophytic trees with broad canopies (Phoenix, USA)	Near-surface temperatures (2 m Ta) and Tmrt for outdoor thermal comfort	Chow and Brazel (2012)
Assessing heat mitigation strategies	Greenery and water bodies (Portland, Oregon, USA)	Tmrt, Ta, globe temperature	Taleghani et al. (2014)
Evaluating the cooling impact of trees and cool roofs in different landscaping strategies (mesic, oasis, and xeric)	Cool roofs and urban forestry and (Phoenix, Arizona, USA)	2 m Ta	Middel et al. (2015)
Assessing effectiveness of UHI mitigation strategies	Grasses, shrubs and trees, application of enhanced albedo substances in outer building surfaces and urban inland water bodies (London, UK)	2 m Ta	O'Malley et al. (2015)
Investigating spatial and temporal pattern of the UHI intensity and evaluate vegetations and cool roof for managing UHI using WRF	Green vegetation and cool roof (Singapore)	Temperature of near-surface air and surface skin	Li and Norford (2016)

WRF is a non-hydrostatic regional climate model (RCM) which is popular for urban meteorological studies (e.g., Imran et al., 2018). WRF-SLUCM has intrinsic constraints for representing buildings in the model, such as extensive (depth < 150 mm) against intensive (depth > 150 mm) roofs and pitched against flat roofs. These might have varying impacts on the surface energy balance, which are difficult for the model to sort out. Different resilience scenarios (expansion of urban green zones and deployment of cool green and white roofs) were simulated with WRF coupled with SLUCM, using a projected heatwave arising in Porto metropolitan zone from 24 to 26 July 2049 (Carvalho et al., 2017). Cool roofs were found to be the most effective in mitigating high urban temperatures, whereas white roofs were considered an economically attractive option. The WRF model was applied at a high-resolution (300 m) and at the city scale for a combined investigation of the urban heat island effect and the feasibility of management strategies, such as of cool roofs and green vegetation (Li and Norford, 2016). The results revealed that the installation of cool roofs at city scale can remarkably bring down the temperature of air near-surface and the skin surface throughout the daylight time (particularly during mid-day) with minor effects after evening. However, green vegetation cover can decrease the temperature of near-surface air beyond 1 °C in the night when the UHI strength is elevated. Many others (e.g., Lee et al., 2016; Crank et al., 2018) applied ENVI-met and WRF-SLUCM to evaluate the effectiveness of NBS (urban parks, roadside plantations, urban green space, green roofs, green walls etc.) in reducing heat stress.

An hourly Surface Energy Balance Model was engaged for a long time series (1981–2014) to simulate the UHI effect in five different sites in Milan, Italy (Mariani et al., 2016). The study also manifested the importance of soil water reservoirs in urban green areas to enhance the cooling impact of urban greenery on UHI by both, replacing sensible heat fluxes with latent heat and by the addition of tree canopy shading. The outcomes of different heat management strategies depend considerably on the particular urban scenario, the confined climatic set-up, and also on the time of the day. This was studied by Saneinejad et al. (2014) who modelled three UHI mitigation measures, i.e. evaporative cooling, albedo enhancement and shading using a microclimate simulation model consisting of three integrated and interconnected sub-models (CFD; Building Envelope Heat and Moisture, BE-HAM, and radiation). They discovered that shading provides the highest cooling, determined according to the Universal Thermal Climate Index for an average summer climate and for heatwave conditions.

### 3.4 Landslides

Table 4 summarises the input variables used to understand landslide hazards, the types of NBS, and the most used numerical models to simulate the efficiency and performance of NBS against landslide hazards. Along with the numerous studies addressing root reinforcement of soils experimentally, models have been developed to represent the mechanical behaviour of roots and their spatial distribution (e.g., Schwarz et al., 2013; Schwarz et al., 2010; Pollen and Simon, 2005). These models are emerging to assess both the mechanical and hydrological effect of NBS against landslides (e.g., Arnone et al., 2016a; Gonzalez-Ollauri and Mickovski, 2017a). Mechanical strengthening of soil by roots increases the soil resistance to shear stress and reduces the risk of landslides. Several studies applied the Root Bundle Model (RBM) (Schwarz et al., 2010; Moos et al., 2016) to quantify area-wise root tensile strength as a function of movement, which had not been considered in previous models (e.g., Wu et al., 1979). The RBM requires the root distribution of the desired plant species, the location of stems, and the stem diameter at breast height (DBH) as input data. The resulting map shows the spatial pattern of root reinforcement which enhances slope stability. This result can be used as a performance indicator for NBS targeting root reinforcement, e.g., comparing the stabilising effects of various tree species (e.g., Chiaradia et al., 2016), different forest stand structures and management practices (e.g., Dazio et al., 2018; Moos et al., 2016) and forest clearing scenarios. The latter includes the decay of root reinforcement following timber harvesting or the rejuvenation after a forest fire (e.g., Vergani et al., 2016, Vergani et al., 2017). Schwarz et al. (2012) investigated the spatial distribution of root reinforcement in a landslide triggering experiment in a mixed forest dominated by ash (*Fraxinus excelsior* L.) on a slope near Rüdlingen (Switzerland). They assessed root diameters and their distribution in soil profiles and through the escarpment of the induced landslide. Based on the collected data, the authors established an RBM for quantifying root strengthening as a function of the trees' stem DBH and the distance from the stem. Other numerical models (Table 4), such as NHWAVE, FUNWAVE-TVD, SSHV-2D (Fornaciai et al., 2019; Emadi-Tafti and Ataie-Ashtiani, 2019), *tRIBS-VEGGIE* (Eco-hydrological) model (Arnone et al., 2016b) and BROOK90 (Federer et al., 2003) have also been applied to evaluate NBS (re-vegetation of shrubs and trees, and their root reinforcement, stabilising and hydrological effects) implemented against landslides. The hydro-mechanical effect of vegetation on soil reinforcement can be modelled by merging the mechanisms by which plants contribute to regulate the hydrological cycle (e.g., evapotranspiration, rainfall partitioning, preferential flow below ground etc.) with variables quantifying the soil stress (Gonzalez-Ollauri and Mickovski, 2017a, Gonzalez-Ollauri and Mickovski, 2017b). In this regard, Plant-Best is a numerical model that considers the hydro-mechanical effect of vegetation on slope stability using easy-to-measure climatic, edaphic and plant metrics to support plant species selection for slope protection using a spatially distributed approach (Gonzalez-Ollauri and Mickovski, 2017a). However, models still need to simulate robustly how plants regulate the hydrological cycle in a context of landslide prevention (e.g., Gonzalez-Ollauri et al., 2020) and how the hydrological regimes in the soil regulate the mechanical response of vegetation (e.g., Gonzalez-Ollauri and Mickovski, 2017c).

**Table 4:** Overview of input variables and models used to simulate the efficiency and performance of NBS against landslides.

Purpose/summary	Type of NBS (place)	Models to simulate NBS efficiency	Input parameters	References
Modelling the spatial pattern of root reinforcement	Re-introduction of vegetation (New Zealand)	Root Bundle Model	Root distribution data, tree stem diameter at breast height	Schwarz et al. (2010) Schwarz et al. (2016) Vergani et al. (2014)
Modelling landslide susceptibility for predicting sustainable forest management in an altered climate	Forest management (Queets watershed within the Olympic Experimental State Forest (OESF)) in western Washington State (U.S.)	Process-based hydrology model (Distributed Hydrology Soil Vegetation Model (DHSVM))	Historic Meteorological Inputs, DEM (150 m), soil and land cover distribution, projected meteorological inputs from climate change scenarios, Soil and vegetation information (cohesion, unit weight), High resolution DEM (10 m)	Barik et al. (2017)
Modelling the effects of sand-filled ditches on the hydrological conditions in a fruit farm on a slope (amount of infiltrating water)	Sand-filled drainage ditch (Olszanka, Poland)	FEFLOW	Slope geometry, ditch dimensions, soil parameters, vegetation cover data	Widomski et al. (2010)
Assessing of the impacts of European forest types on hill slope stabilisation (mountainous area of Lombardy, Italy)	Forest Management	Limit equilibrium model, probabilistic framework (Monte Carlo techniques)	Root density and root mechanical properties	Chiaradia et al. (2016)
To estimate the function of vineyards on slope stabilisation by modelling the additional strengthening to the soil supported by grapevine roots and their spatial distribution.	Plant roots and vegetations (northeastern part of Oltrepo Pavese, Northern Italy)	Root Bundle Model, Slope stability model	Root distribution and characteristics (diameter, length etc.), soil strength parameters	Cislaghi et al. (2017)
Evaluate the impact of underlying foundations of birch trees on soil fortification and slant adjustment.	Birch trees	PLAXIS	The rainfall, slope gradient, geotechnical and hydrological parameters and soil thickness	Lotfalian et al. (2019)
To investigate the capacity of vegetational NBS to reduce the onset and propagation probabilities of tsunamis generated landslides at Stromboli Island, Italy	Trees, forests, and grasslands	FUNWAVE-TVD	Bathymetric, topography	Fornaciai et al. (2019)
To investigate the different aspects of hydrological and greenery effects on the stabilisation of hillslopes	Hydrological and greenery	SSHV-2D	Bathymetric, topography	Emadi-Tafti and Ataie-Ashtiani (2019)
To simulate the efficiency of species and assessing its mechanical resistance against shallow landslides.	Vegetation	tRIBS-VEGGIE	Bathymetric, topography	Arnone et al. (2016a)
To simulate the effectiveness of NBS against shallow landslides.	Forest canopies, leaf area index and plant height, and optimised forest management	BROOK90	Forest structure, meteorological variables, root density, hydrological parameters and soil permeability	Federer et al. (2003)

PLAXIS is a 2D and 3D numerical model for the simulation of soil deformation and stability, which can be used to analyse the efficiency of NBS for stabilising slopes and reducing landslide risk (Cofie et al., 2000). The rainfall, slope gradient, geotechnical and hydrological parameters and soil thickness are the main forcing parameters. Lotfalian et al. (2019) used this model to evaluate the impact of underlying foundations of birch trees on soil fortification and slant adjustment. The slant solidness found to be the function of soil varieties and the age of the tree. The effectiveness of the NBS (trees) in delaying the risk of landslide increased with the age of the tree (e.g., from 7 to 15 years).

The innovation and developments introduced in non-hydrostatic 1D, 2D and 3D NHWAVE model (Ma et al., 2012) and completely nonlinear diffusive Boussinesq long-wavelength FUNWAVE-TVD model (forced with the NHWAVE outcomes), have made them capable of simulating tsunami generated landslides along with the potential vegetation NBS (trees, forests, and grasslands) to reduce the associated risk (Shi et al., 2012). Fornaciai et al. (2019) investigated the capacity of vegetational NBS to reduce the onset and propagation probabilities of tsunamis generated landslides at Stromboli Island, Italy, by using bathymetric and topographic datasets as input variables.

SSHV-2D is a 2D hillslope stabilisation model which simulates hydrological and greenery effects. Emadi-Tafti and Ataie-Ashtiani (2019) applied the SSHV-2D model to investigate the different aspects of hydrological and greenery effects on the stabilisation of hillslopes. The analysis showed that matric suction in the unsaturated zone and the presence of high-density trees on the slopes enhance the safety factor by more than 90% and up to 50%, respectively.

tRIBS-VEGGIE is an eco-hydrological model (Lepore et al., 2013; Arnone et al., 2016a). It offers a comprehensive impact evaluation framework of greenery on the stabilisation of hillslope by simulating soil water availability due to moisture uptake by roots, the process of evapotranspiration, and leaf interception. Weather parameters (wind speed, vapor pressure, precipitation, shortwave radiation, atmospheric pressure, evapotranspiration, cloudiness, and air temperature) and leaf area index are the main input parameters. Arnone et al. (2016a) used this model to assess the stability enhancement achieved by different vegetation species, mainly dependent on their root depth and to determine the efficiency of NBS in reducing the intensity and magnitude of shallow landslides.

BROOK90 is a comprehensive and process-based hydrological model that simulates evapotranspiration, vertical soil water motion and river discharge at the micro-scale (Federer et al., 2003). The model's main forcing parameters are forest structure, meteorological and hydrological variables, root density and soil permeability. In this model, the characteristics of NBS (forest canopies) that are used to simulate their effectiveness against landslides are the year-round leaf area index and plant height, and optimised forest management practices.

### 3.5 Storm surges and coastal erosion

The efficiency of NBS for storm surges and coastal erosion can be simulated using several numerical models and input variables, as indicated in Table 5. For example, the SWAN model assumes that the propagation of wave energy is attenuated by green cover due to the impact of the waves on the vegetation, where the latter is modelled as a vertical element. The model is sensitive to the shape of the frequency spectrum along with the directional dissemination of the waves, whereas the schematisation of the vegetation layer is also important and has to be factored in. The Suzuki et al. (2011) model can calculate 2D wave dissipation over a vegetation field including wave breaking and diffraction. This model can be applied to a field scale of NBS but requires input data on the wave geometry, direction and frequency, as well as the geometry and type of the NBS. This model has been validated against experimental data but has not been applied in the design of NBS or estimation of the effectiveness of existing NBS. The *XBeach model* is capable of simulating flow, waves, sediment transport and coastal morphological changes (Roelvink et al., 2009) in scenarios that include NBS, such as mangroves, sea grass, coral reefs, etc. This model is usually applied to medium-scale study areas (few km) using short simulation periods (hours to days). It is based on the same general assumption as the SWAN model and requires wave characteristics, bathymetry and vegetation descriptors as inputs. The limitations of the model include the lack of 3D consideration of the NBS at the field scale and verification in the design and monitoring of new NBS.

**Table 5:** Overview of input hydroclimatic variables used to understand storm surge risk and models used to simulate the efficiency and performance of NBS against storm surges.

Purpose/summary	Type of NBS (place)	Models to simulate NBS efficiency	Input hydroclimatic indicators	References
Quantifying the reduction of wave height and wave energy;	Salt marsh (laboratory)	The rate of wave height decay diminishes with distance into the marsh	Sea level	Hadadpour et al. (2019)
Quantifying the reduction in flood/wave velocity; minimising net sediment loss	Salt marsh (Chesapeake Bay; USA)	Relative reduction in flood/wave velocity; net sediment loss; Water level attenuation rates	Vegetation type, density, distribution; water pressure; topography; current profile during storm; wave velocity	Paquier et al. (2017)
Quantifying the stability of a marsh	Salt marsh (Alabama, USA)	SWAN	Significant wave height; frequency of occurrence of significant waves	Roland and Douglass (2005)
Explore the effect of a mangrove island on waves reaching port, which lies behind the island; explore the effects of eco-engineering and managing mangroves for coastal risk reduction	Mangroves (forests) (Kanika Sands mangrove island, Orissa, India)	SWAN model	Significant wave height; frequency of occurrence of significant waves, distance to port, type of mangrove trees, extent of mangrove forest	Narayan et al. (2010) Hoque et al. (2020)
Quantify the effect of mangroves on storm surge peak water levels; the effect of wind waves and ground slope;	Mangroves (Mathbaria, Bangladesh)	1D nonlinear, long wave differential equation	Maximum wind speed; water levels	Tanaka (2008)
Explore the effects of land cover types on flood extent.	Mangroves (Biscayne Bay, Florida, USA)	Unstructured Eulerian-Lagrangian Circulation (ELCIRC) model	Peak wind speed 227 km/h, maximum storm tide 5.2 m; Coastal mangrove zone 1 to 4 km wide with tree heights of 1 to 20 m, species (*Rhizophora mangle*, *Avicennia germinans*)	Xu et al. (2010)
Quantify peak water reduction through NBS area	Mangroves (Gulf Coast, Florida, USA)	Coastal and Estuarine Storm Tide (CEST) model	Maximum winds of 195 km/h speed, peak water level 5 m. Dominant species *R. mangle*, *Laguncularia racemosa*, *A. germinans*. Trees 4 to 18 m high, stem diameters 5 to 60 cm. Mangrove width 6 to 30 km; recorded water levels	Zhang et al. (2012) Liu et al. (2013)
Quantifying the reduction of wave loading, flood/wave velocity	Vegetated berms (similar to dunes), Henderson Point, Mississippi, USA	Coupled storm surge and wave model (ADCIRC and SWAN) Hydrodynamic model (XBeach)	Sea levels high water mark elevation records, ground surface elevations (digital terrain model, LIDAR), flood hazard maps, storm return periods, 1:100 flood elevation, future sea level rise, storm locations, winds, pressures; vertical/horizontal wave loading; hydrodynamic force of drag, current velocity. Exposure, aspect and water availability considered for the vegetation on the berms	Web et al. (2018)
To model the wind field which drives the storm surge	Any/none (coastal USA)	Sea, Lake, and Overland Surges from Hurricanes model (SLOSH model)	Estimate storm surge heights resulting from historical, hypothetical, or predicted hurricanes by taking into account the atmospheric pressure, size, forward speed, and track data. A set of physics equations which are applied to a specific locale's shoreline, incorporating the unique bay and river configurations, water depths, bridges, roads, levees and other physical features	Glahn et al. (2009)
To quantify the benefits from reef management.	Coral reefs (Global)	Nearshore hydrodynamics reef wave model, nearshore hydrodynamics total water level model, model of wave setup and run-up	Coastal profiles (2 km resolution), global wave climate and sea levels, topo- and bathy-metric data,	Beck et al. (2018)
To quantify coastal region resilience and protection offered by three types of NBS.	Reefs, seagrasses, and mangroves (Belize)	Numerical model for wave evolution and storm surge	Present and future scenario for non-storm and storm conditions; sea level rise, coral reef scenarios (live, decreasing, no corals); seagrass scenarios (different drag coefficient); mangrove conditions (presence of mangroves, drag coefficient)	Guannel et al. (2016)
To quantify the flow characteristics and sediment trapping capacity of seagrass meadows.	Seagrass meadows (laboratory)	Experimental model of the flow characteristics and sediment trapping capacity	Numerical model with measured shear stress and turbulence of flow, leaf density	Hendricks et al. (2008)
To evaluate the effectiveness of coastal wetlands in reducing expected flood damages.	Coastal wetlands, USA	Regression analysis	Wind speed, storm tracks and frequency of 34 major US hurricanes since 1980	Costanza et al. (2008)
To assess future coastal flood risk in the Gulf of Mexico coast, USA.	Wetland restoration Barrier island restoration Oyster reef restoration Beach restoration US Gulf of Mexico coast	Open-source software ‘CLIMADA’, and its ‘COASTAL’ module	The pressure, wind, rainfall, wind-waves and storm surge were calculated using parametric models.	Reguero et al. (2018)

The Sea, Lake, and Overland Surges from Hurricanes (SLOSH) model, developed and applied by the NOAA Hurricane Center (https://www.nhc.noaa.gov/surge/slosh.php), is an excellent example of why models are necessary to assess risks associated with HMHs in general and of storm surges in particular. The storm surge interactive risk maps of NOAA National Hurricane Center's Storm Surge Unit (https://www.nhc.noaa.gov/surge/faq.php#2) show potential storm surge impacts for all areas and incorporate varying landfall locations, local bathymetry and topography, varying storm sizes, forward speeds, tracks, approach angles, and tide levels. This is accomplished by performing thousands of different SLOSH simulations for a given area and then compositing the results into a worst-case snapshot, indicating storm surge vulnerability. Thus, for a given area of interest, the storm surge interactive risk maps make use of thousands of hurricane landfall scenarios. The maps are operationally used in support of interventions to mitigate risks and impacts of storms.

The TELEMAC model is a finite element computer-based model developed by the Laboratoire National d'Hydraulique et Environnement. The 2D module within is capable of solving the shallow water equations, while the 3D module solves the complete Navier-Stokes equations, which govern the wave dynamics. TELEMAC can be used for the detailed modelling of the effects of NBS on coastal erosion. While validated against real-life tidal hydrodynamics at an estuary scale (Stark et al., 2016), the use of this model in the planning and construction of NBS, however, has not been reported in the literature.

The ADCIRC model is a finite element model based upon the solution of the wave continuity equations (Luettich et al., 1992). It is capable of accounting for different seabed drag coefficient formulations, as well as changes in land cover, wind and atmospheric pressure which makes it ideal for NBS applications. This model is usually coupled with wind wave models, such as SWAN (Suzuki et al., 2011; Roland and Douglass, 2005) for application in wave propagation, storm surge modelling and damage assessment (e.g., Highfield et al., 2018).

The Maritime Forest Model is a semi-analytical model of wave propagation through a lattice-like array of vertical cylinders which simulate vegetation planted as part of an NBS solution (Mei et al., 2014). The model was validated against laboratory experiments with analogue vegetation placed in a flume. Focusing on micro-scale wave propagation and flow around rigid ‘vegetation’, this model requires the macro scale solution of the wave propagation before it can be applied on realistically-modelled vegetation.

## 4 Advantages and limitations of modelling techniques

Numerical models (hydrological, hydraulic and aerodynamics) are the most frequently used methods to assess NBS effectiveness for HMR mitigation. The strongest benefit of numerical models is their capability to bring important information about state variables (e.g., flow depth) into the simulation via the use of data assimilation of non-conventional parameters and/or appropriately formulated dynamic boundary conditions. The major advantages, limitations, possible uses and upgrades of these models are presented in Table 6. For instance, they are capable of simulating individual NBS in a detailed manner; need no spatial discretisation and have no or little trouble with mass conservation or spatial diffusion. They are also directly associated with NBS attributes, HMR mapping, predicting and scenario analysis of NBS performance and efficiency (e.g., MIKE-SHE). Eulerian and Lagrangian-based 3D numerical models, such as TUFLOW, TELEMAC, MIKE-SHE have advantages over conventional mesh or grid-based approaches in their capacity of simulating severe weather conditions, e.g., rapidly varying flow, tsunami, and tidal waves. However, they suffer from a few limitations including: (i) propagation of input errors in time, (ii) high computational cost, and (iii) high data requirements (Fig. 3). Some models, such as SWMM, XBeach, TELEMAC, LISFLOOD-FP, Flood Modeller, FUNWAVE-TVD, ENVI-met have great potential in evaluating NBS detailed performance at the local scale but they can neither simulate the HMH routing nor their generating processes (Zhang et al., 2018; Zhang and Chui, 2017). Other models (e.g., MIKE-SHE, SWAT, HEC-HMS/RAS, ParFlow-TREES, ACRU, SIMGRO, and WRF-SLUCM) can simulate the generation of HMHs and be applied at the watershed scale (Hoghooghi et al., 2018; Barron et al., 2013; Locatelli et al., 2017; Trinh and Chui, 2013). Not all numerical models operating at the catchment scale are flexible to operate at smaller spatial scales, e.g., local urban scale to simulate the performance of urban plants within sustainable urban drainage systems (SuDS).

**Table 6:** Outline of the advantages and limitations of numerical models that can evaluate NBS against different HMHs.

Models	Advantage	Disadvantage	Potential application	Potential improvement	Reference
Surface/subsurface models, e.g., • MIKE-SHE • SWAT • MODFLOW • WRF • HEC-RAS • ParFlow-TREES • ACRU • SIMGRO	• Incorporate almost all relevant surface and subsurface hydrological process • Can simulate large-scale NBS planning due to normally great capacity in representing the variations of processes and features spatially	• Coarse in spatial and temporal resolutions • Cannot simulate urban drainage systems (UDS) • Cannot simulate the detailed geometry and design features of NBS • Data intensive	• Evaluate the performance and efficiency of NBS against HMHs in the catchment scale • Evaluate the optimal allocation of NBS in the catchment scale • Evaluate the performance and efficiency of NBS against HMHs in the catchment scale • Evaluate the optimal allocation of NBS in the catchment scale	• Allow finer spatial and temporal resolutions • Develop built-in NBS modules to evaluate NBS more flexibly • Coupling with hydraulic modes to simulate UDS.	Ewen et al. (2000) DHI (2007) Kim et al. (2008) Markstrom et al. (2008) McKane et al. (2014)
Green NBS-specific surface e models, e.g., • SWMM-LID-GW • SWMM • GIFMOD • ENVI-met • FUNWAVE-TVD • SSHV-2D • tRIBS-VEGGIE • BROOK90 • SWAN • ADCIRC • XBeach	• Can simulate and evaluate the green-NBS and SUDS at catchment scale • Power in runoff simulation and routing • Considers two-way interaction between green NBS and groundwater • Flexible to simulate NBS design and performance evaluation	• Does not simulate groundwater flow and requires groundwater data as direct input • SWMM and SWINGO-VFSMOD can only simulate an individual NBS by assuming homogeneous soil profile • Cannot represent some design feature of NBS and their efficiency evaluation		• Coupled with a subsurface hydrological model • Improvement to consider multiple NBS in SWMM and SWINGO-VFSMOD and overlap groundwater module • To enable more flexible NBS designs and evaluations	Massoudieh et al. (2017) Zhang et al. (2018) Roldin et al. (2013) Locatelli et al. (2015) Fox et al. (2018)
Variably saturated models, e.g., • LISFLOOD-FP • HYDRUS • Flood Modeller • PLAXIS • TELEMAC	• Accurately simulate subsurface flows • Easily track and visualise subsurface flows • Flexible to simulate the detailed geometry of NBS	• Simplify the simulation of surface rainfall runoff generation processes • Cannot simulate large scale NBS designing and planning		• Coupled with a subsurface hydrological model	Hsieh et al. (2000) Diersch (2005) Simunek et al. (2005)

The consideration of both spatial and temporal scales is essential for the accurate operationalisation of NBS and the connectivity between multiple NBS, the environment and the human communities in which they are installed (Kumar et al., 2021). The spatial and temporal context of NBS has not been properly addressed, but it is unquestionable that aspects related to space and time must be considered (Raymond et al., 2017a; Ruangpan et al., 2020). Haghighatafshar et al. (2018) categorised the spatial scale of NBS implementation into four types: (i) microscale, (ii) watershed/mesoscale, (iii) macroscale/regional-scale and (iv) continental/megascale. As a result, the spatial scale to evaluate the performance and impact of an NBS varies with the type of NBS and with the type of impact considered (see Kumar et al., 2021). The spatial scale establishes context boundaries, and it determines the size of the NBS action, e.g., landscape, catchment, stand, or plot scale (e.g., Bock et al., 2005). Moreover, some HMHs can only be perceived at a given spatial and temporal scale (e.g., landslides – local or landscape scale and slow-moving; floods – mesoscale catchment scale and fast-moving), and recurring HMHs may require greater efforts to devise flexible and resilient NBS. The performance of some NBS against HMHs will only be perceptible within a given time scale, e.g., plant based NBS against landslides and erosion will only be fully functional when a dense vegetation cover has established on the NBS (Morgan, 2014).

Temporal scale over which a specific NBS becomes fully operationalised and effective is not widely available in the scientific literature as it varies across HMRs, selected NBS and their location. Monitoring and evaluation can be done each hour, day, week, month or yearly depending upon the problem being faced, its priority, NBS design and agreed goals (Kumar et al., 2021). Temporal scale can be categorised into (i) short (within 5 years), (ii) medium (5–10 years) and (iii) long-span (over 10 years) (Raymond et al., 2017a; Kumar et al., 2021). In the socio-economic context, the temporal and spatial scales will be a factor in the priorities and perceptions of stakeholders. Numerical models can help overcome the challenge of integrating the scale within NBS projects throughout their life cycle. For example, spatially distributed, numerical models with the ability of processing time series and forecasting while dealing with uncertainty (e.g., Gonzalez-Ollauri and Mickovski, 2017a, Gonzalez-Ollauri and Mickovski, 2017b, Gonzalez-Ollauri and Mickovski, 2017c) can be very useful to envisage the performance of pools of NBS at multiple scales and under multiple socio-ecological scenarios. However, numerical models are sometimes not flexible enough to handle multiple spatial and temporal scales. To overcome this issue, access to the model code is essential, facilitating the integration of scale and models into open-access programming languages and software (e.g., R, QGIS; e.g., Gonzalez-Ollauri and Mickovski, 2017a, Gonzalez-Ollauri and Mickovski, 2017b, Gonzalez-Ollauri and Mickovski, 2017c). Overall, the potential shortcoming of all the numerical models presented in this paper is that the ecosystem services of NBS are not fully encompassed in their governing equations or physical principles (e.g., conservation of momentum, mass, and energy). The interaction and feedback loops are missing between numerical models and full functions of biodiversity and ecosystems, which portray the ecosystem's ability to produce multiple services/benefits.

### 4.1 Floods

HEC-RAS, MIKE-SHE, SWMM, LISFLOOD, HYDRUS and SWAT are the most widely used numerical models to simulate and evaluate the flooding scenarios. Each modelling technique has its own advantages and limitations. For instance, the HEC-RAS model can perform a sensitivity and scenario evaluation to identify the important model parameters for a chosen study and can accurately replicate the observed water surface with and without NBS in place (Ardıçlıoğlu and Kuriqi, 2019). The major disadvantage of HEC-RAS is that the model performance deteriorates considerably to evaluate the performance of NBS in situations where the channel geometry becomes complex, such as varying cross-sectional area and frequent change in channel direction (Papaioannou et al., 2017). The MIKE-SHE model is a comprehensive tool for the simulation of floods and droughts along with the corresponding NBS at the catchment scale. However, the application of this model is hindered by coarse spatial and temporal resolution, extensive data requirements and cannot simulate the detailed geometry and design features of NBS at local scale, which limits its application in urban areas. The SWMM model is effective in simulating runoff and flooding generated from a single rainfall event in urban areas, where the model output can be generated in minutes or hours time scale (flash and urban floods). It accounts for various hydrologic processes that generate and reduce runoff from urban regions (e.g., runoff reduction through NBS (grey-green) practices, time-varying precipitation, and infiltration of rainfall into unsaturated soil layers). Therefore, it is an advantageous and efficient tool to evaluate grey measure (e.g., pipes and storm drains) with NBS to create cost-effective hybrid (grey-green, Section 3.1) NBS for flood risk management. The main flaw is that, in the situations where the model needs to run for longer time period, the computation time required to simulate runoff may range from several hours to days depending on the size of the hydrological system which could cause computational difficulties (time and complexity) (Burger et al., 2014). The other models require high resolution digital elevation model data of the river, its floodplain and the planned NBS (e.g., LISFLOOD-FP).

The strength of HYDRUS model is simulating the movement of water and solutes in saturated/unsaturated soil medium and evaluating the impact of vegetation (green-NBS) on the soil moisture, which is a critical parameter for flood modelling. It can generate output at seconds time scale as well as for a long-time frame (Simunek et al., 2005). However, the main problem with the HYDRUS model is that it can only simulate the water/solute movement for a limited number of hydrological systems, where the model substantially simplifies the complexity of a real-world system (Venkatraman et al., 2014). SWAT can successfully analyse water, sediment and agricultural chemical yields in large and complex watersheds at daily time scale and for a longer time period corresponding to various climate and terrain characteristics (Shi et al., 2011). However, the model requires a set of basins and weather-related data as an input. Assembling the input database for running the SWAT model requires considerable time and effort (Jayakrishnan et al., 2005). Furthermore, since the model was primarily developed to simulate runoff at river basins in the USA, application of SWAT model for basins located outside the USA needs to be calibrated.

### 4.2 Droughts

For droughts, SWMM does not accurately simulate the interaction between unsaturated and saturated flows because it linearises the soil water holding curve and ignores the influence of underground water on the NBS to maintain deep ground/subsurface water flows (Zhang et al., 2018). To partially resolve these limitations, Zhang et al. (2018) created a module that increases the hydraulic connectivity of unsaturated and saturated flows and the relation between NBS and groundwater flows in SWMM. They validated the improved version of SWMM by simulating the performance of different green approaches (e.g., porous pavements, bioretention cells) in surface and subsurface groundwater settings. Nevertheless, it could not mimic groundwater changes but needs the direct forcing of underground water tables, which significantly hampers its potential implementation.

ACRU model's ability to simulate the different hydrological components enables associations with the economic implications of water management (Rebelo et al., 2015). It efficiently mimics subsurface groundwater changes but has drawbacks when deep regional groundwater exists (Mander et al., 2017). The ACRU model is more advantageous in data-sparse areas because it is less data intense compared to other process-based distributed or semi-distributed models and its variables are directly quantifiable on-site. The key disadvantage for its application in many areas is that its standard parameters were adjusted soil data from Southern Africa (Schulze, 1995).

The main strength of the SIMGRO model is its integrated system approach. It can simulate the interaction among soil water, plants, meteorological conditions, groundwater and surface water. SIMGRO can be applied in circumstances where varying situations influence several components of the hydrological cycle. For example, the model will forecast the impact of changes in shallow and groundwater distribution or a change within drainage networks on the crop irrigation (Querner et al., 2008). The model is not characterised for its simplicity, which constitutes its main disadvantage.

### 4.3 Heatwaves

ENVI-met is one of the best models to evaluate NBS effectiveness against heatwaves at the microscale. One of its main advantages is the ability to evaluate the thermal effects of buildings, vegetation and land cover (Égerházi et al., 2014). However, the model has shown some stability issues in abutting neighbourhoods or winding urban canyons, non-prognostic simulation of RH (Bruse and Team, 2020), scale and computational time. For example, upscaling from microscale to watershed scale is not feasible as it cannot flexibly simulate the NBS geometry. Recent developments improved the definition of the forcing parameters, but the impact of the greenery on longwave radiation fluxes were ignored (Égerházi et al., 2014; Bruse and Team, 2020). Using only one input data for each weather parameter, can simulate ambient temperature, RH, solar radiation, and wind speed but only for a maximum of one to two weeks. WRF, another major model for assessing heat relieving NBS, has been broadly utilised to simulate the urban climate. However, WRF suffers from the coarse categorisation of urban canopy parameters, and further upgrades would require considerable effort (Zhang and Chui, 2020).

### 4.4 Landslides

RBM, FUNWAVE-TVD, BROOK90, PLAXIS, SSHV-2D, and tRIBS-VEGGIE were reviewed to evaluate performance and efficiency of NBS used against landslides in Section 3.4 and here for the sake of brevity, the advantages and limitation can be discussed only for RBM, FUNWAVE-TVD and BROOK90 models. The RBM is suitable to realistically represent the soil reinforcement of roots and is based on the dynamic relationship of the roots' tensile strength against displacement (Schwarz et al., 2010; Moos et al., 2016). The model considers the maximum reinforcement as a function of the distance to a tree stem. With mapped tree locations and their diameter at breast height, the RBM can be efficiently used to estimate root reinforcement area-wide and to consider its spatial variability for slope stability modelling. However, several limitations go along with the RBM, including the assumption of isotropic root growth, which may not be justified in tree stands with competing individual trees. Also, the actual distribution of roots on slopes may disagree with this assumption. Nevertheless, the RBM is feasible for estimating root reinforcement at the stand and slope scale rather than for individual shallow landslides.

FUNWAVE-TVD is a well-balanced conservative form of the governing equations developed to facilitate the hybrid numerical scheme. This is an advantage of using this model to treat wave breaking as shock waves by switching the Boussinesq equations to nonlinear shallow water equations when the ratio of surface elevation to local water depth exceeds a certain threshold. FUNWAVE-TVD has substantial benefits due to the small number of terms in both continuity and momentum equations and precisely simulates the propagation of fully dispersive water waves and the efficiency NBS (e.g., vegetations). However, waves are generally produced and start their journey in deep waters while the depth limitation of the FUNWAVE-TVD models still cannot cover properly the entire domain from shore to the deep ocean when dealing with real life sea states (Liu et al., 2020).

BROOK90 is a relatively complex process-orientated model that can generate daily evapotranspiration values separated into transpiration from vegetation, direct evaporation from the soil and vaporisation of intercepted rainfall from vegetation. It requires only a few daily input data, such as precipitation and minimum and maximum temperature once the model is calibrated. All data are freely available from a number of networks for collecting and sharing data (e.g., FluxNet and MODIS data). The limitations are that the model does not consider non-green leaves which may intercept solar radiation and precipitation but do not transpire. Similarly, some parameters, such as variation in albedo and lateral transport of water to adjacent pixels as well as channel routing is not presented (Federer et al., 2003; Schaffrath et al., 2013).

### 4.5 Storm surges and coastal erosion

To improve the current modelling approach for the evaluation of NBS in reducing the storm surges and coastal erosion risk, Spalding et al. (2014a) suggested holistic coastal protection planning models where the NBS would be combined with the traditional infrastructure into a single integrated planning framework. In this regard, the existing micro and macro-scale numerical models can be improved to account for NBS with the addition of parameters relevant to vegetated infrastructure and they can become more accessible and easier to use by communities and local decision-makers. These models should be a purpose- and site-specific, cover the meso-scale (e.g., cross- and long-shore extent of the NBS) and include components of engineering and ecological stability assessment. Overall, better parameterisation of the relationships between vegetation characteristics and wave forces (trying to uproot the mangrove or overturn oyster reefs) can be achieved if knowledge from anchorage mechanics and geotechnical engineering is translated into the NBS design. Case studies on NBS for storm surges and coastal erosion outside the tropics are needed to assess the range of numerical models available at a global scale.

## 5 Economic evaluation of NBS

**Fig. 4:**
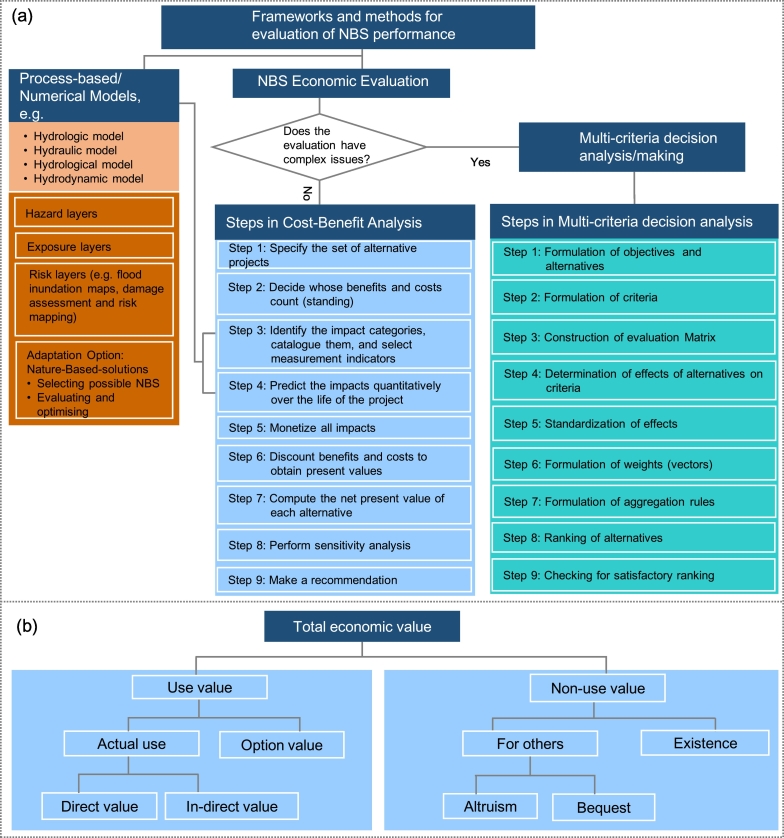
(a) Nine steps approach to CBA to assess the worthiness of EU funded projects and review cost-effectiveness comparison between NBS and grey approaches (source: Boardman, 2014). (b) Total economic value of an NBS service with its different value components (source: Turner et al., 1994).

In addition to bio-geophysical modelling, socio-economic models and analysis tools, such as the CBA are often used to appraise NBS designs. CBA is an evidence-based analysis framework that can be used to evaluate the monetary value of a given project, have better alternative options, to strengthen and make the decision-making processes transparent and help select appropriate NBS and rational resource allocation for every major project (Sartori et al., 2014). Boardman's (2014) nine steps of CBA (Fig. 4a) is one of several standardised CBA structures to assess the worthiness of any projects (Sartori et al., 2014) and review cost-effectiveness comparison between NBS and grey approaches. Life cycle costing is one exemplary cost-benefit perspective where cost of construction, operation and maintenance Nordman et al. (2018), along with return on investment (De Risi et al., 2018) are considered for the entire project life. The information regarding the type of solution (grey versus NBS), their benefits, costs and indicator-based impacts within the life cycle of the project (Fig. 1) allows analysts to estimate the monetary value of each impact catalogued. Impact chains from NBS and grey solution interventions can have complex pathways requiring interdisciplinary cooperation to model and describe different, plausible scenarios. Consequently, monetisation of the impacts can be a difficult task requiring assumptions since NBS and grey solutions can have effects in multiple value categories.

Fig. 4b illustrates common typology to divide the total economic value of a commodity such as an ecosystem service into its different value components. This typology reflects what kind of impacts ecosystem services have on the wellbeing of individuals (Freeman, 2003). Use values rise from the actual or planned consumption and exploitation of a service which yields wellbeing. Direct values have a clear market price, such as bare land value of standing forest. In-direct values do not have market price as long as there is no fee, such as entrance fee to a beach. Typically, in-direct value captures wellbeing from recreation. Option values exist when individuals are willing to pay for the option to use ecosystem service in the future. In contrast to consumption, non-use values of ecosystem service increase the wellbeing of individuals without exploiting. Existence value means that individuals receive wellbeing by just knowing that something exists. An example could be a healthy population of species in a natural habitat. Altruistic and bequest values means that individuals are receiving wellbeing by knowing that other individuals have the possibility to use given ecosystem service now and in future (Bateman, 2002). This typology illustrates why valuation of an ecosystem is such a demanding task as the actual use value is the only category where ecosystem value has a market price.

Links between biophysical modelling of NBS and CBA can be found from Fig. 4a (steps 3 and 4) of the Boardman structure, where the economic analysts identify impact categories and predict the impacts quantitatively over the life of the project. Often NBS CBA is a part of an interdisciplinary project where impacts of NBS are biophysically modelled and/or tested. Cooperation between economists and natural scientists can be beneficial in the step when impact categories and indicators are selected to ensure as comprehensive CBA as possible. In practice, CBA analysts should understand from the bio-geophysical modelling what are the expected quantified impacts with and without a given project. In steps 3 and 4, the bio-geophysical modelling outputs, such as exposure, hazard, and risk layers (e.g., flood inundation maps, damage assessment and risk mapping) and the efficient and optimal number of NBS for each HMRs are used to force socio-economic models (CBA). However, we want to point out that the input to the CBA is highly dependent on anticipated impacts. When the impacts from a project implementation have observable market prices, those are direct values whereas for impacts having intangible values, different economic valuation methods such as travel cost methods or contingent behaviour methods are required to evaluate, e.g., social effects (Bateman et al., 2002). The net present value (NPV) for each project alternative is the difference between value of cost and the value of benefit. A project should be recommended if NPV is greater than zero (Pearce et al., 2006) or greater than that for other alternatives (Boardman, 2014).

When social or ecological effects are impossible to monetise, then the project performance is evaluated with multiple non-monetary criteria, such as cost-efficient analysis, cost impact analysis, life cycle cost analysis, natural capital accounting and multi-criteria decision analysis/making (MCDA/MCDM) (Nika et al., 2020; Hassangavyar et al., 2020). The role of non-market valuation techniques or alternative appraisal methods, e.g., MCDA/MCDM is critical in the case of NBS targeting HMRs. The concept of NBS withholds the idea of co-benefits which might be changed indirectly by less tangible values, such as increased biodiversity or recreational value. A holistic evaluation of NBS requires these impacts taken into consideration when the effectiveness is assessed. Cost-effectiveness analysis compares costs of different projects to their corresponding quantified impact which is not monetised, such as number of lives saved (Boardman, 2014; Nyborg, 2012). Furthermore, in the case of highly complex environmental problems, the CBA approach could not provide the best options among the considered alternatives. In this regard, MCDA/MCDM is useful when there are distinctive and clashing criteria to be pondered (Loc et al., 2017; Alves et al., 2018; Loos and Rogers, 2016). MCDA or MCDM is a method to support decision-making processes, by optimising the balance between the advantages and limitations of different alternatives to accomplish a specific goal (e.g., planning and designing of NBS scenarios with multiple objectives). Annema et al. (2015) applied both CBA and MCDM to investigate and discuss a politician's view of how a useful transport policy evaluation instrument might look like. MCDA/MCDM can be more flexible than CBA. In a CBA, analysts must monetise everything, which might be quite limiting and sometimes completely impossible. In MCDA/MCDM one can set criteria which are measured more freely, and the monetising does not restrict the analysis much. It supports in decision making problems, such as selecting the best adaptation options (optimal NBS), policy-decisions, farming-decisions and any other environment and social problems by illustrating the performance of alternatives (based on the evaluation criteria, Fig. 4a), exploring trade-offs, formulating a decision and testing its robustness (Mendoza and Martins, 2006; Hassangavyar et al., 2020; Sironen et al., 2020). It can be used to structure complex problems and help find a better understanding of costs and benefits. Therefore, MCDA is useful for decision-makers when there are multiple and conflicting criteria to be considered (Ruangpan et al., 2020). The MCDA assigns weights to each criterion and produces a ranking of the different measures (Chow et al., 2014; Jia et al., 2015). For instance, Loc et al. (2017) consolidated the outcomes of social surveys and numerical modelling into an MCDA and rated the substitutes elicited from their flood alleviation, pollution removal and aesthetics characteristics. Loos and Rogers (2016) utilised multi-attribute utility theory to judge value of service from each substitute contemplating priority and usefulness as separate and self-contained elements. Petit-Boix et al. (2017) endorsed to integrate risk appraisal models, the economic worth of the anticipated structural and ecosystem loss, and environmental reverberation of NBS for further research. MCDA method requires analysts to set a goal and different predetermined criteria which needs to be fulfilled before the goal can be reached. The aim of MCDA is to evaluate different projects in this framework and how each project meets the criteria (Garfì et al., 2011; Liquete et al., 2016). Sironen et al. (2020) applied MCDA to optimise the policy instrument scenarios/criteria (e.g., enforced spatially concentrated permanent conservation, voluntary permanent conservation, voluntary temporary conservation and voluntary permanent conservation with active nature management) for conserving forest biodiversity in Southwestern Finland. The result showed that minor differences among the instruments, with temporary and voluntary permanent conservation carried out via voluntary efforts producing the maximum total benefit. Furthermore, the MCDM techniques can be used by soil conservation decision-makers to identify areas prone to land degradation at watershed-scale (e.g., Hassangavyar et al., 2020).

### 5.1 NBS-CBA for floods

Foster et al. (2011) compared the price and advantages of grey infrastructure alternatives to the green infrastructure for flood protection. They found that: (i) NPV of green roofs was ~10–14% higher than conventional roofs; (ii) trees provided US$1.3 billion in stormwater profits (based on US$0.66 /cubic foot of storage) in Houston, Texas; (iii) wastewater treatment system using constructed wetlands costed 50% lesser (~US$5.0) per gallon of volume than a traditional treatment provision (US$10.00); (iv) permeable pavement reduced storm-runoff volume by 70–90%, same as meadow or forest; and (v) a rezoning would save nearly 16-times more (US$155 million) than flood control infrastructure (US$10 million) in terms of avoided flood damages to manage probable future climate change impacts in a community in Canada.

Liquete et al. (2016) conducted MCA in Gorla Maggiore, a small township in northern Italy to study the benefits of ecosystem services, i.e. water purification, natural habitat recreation and flood regulation. They compared green infrastructure with traditional grey infrastructure and with past situations (poplar tree plantation). Nurmi et al. (2016) studied the cost effectiveness of green roofs to provide ecosystem services in the city of Helsinki. The study focused on multiple benefits that green roofs provide such as energy savings, storm-water control, scenic and health benefits and emission regulation. The total benefits to cost ratio (B/C) for green roofs in stormwater management was 0.9–2.2 in 50% green-roof buildings scenario and yielded benefits of ~US$2.3 per 4.1m^2^ for the city-wide green-roof scenario.

Narayan et al. (2017) assessed the economic and biophysical significance of the coastal wetlands in the northern USA by performing an ecosystem service analysis using the avoided cost method and found that wetlands prevented nearly US$625 million due to flood damages during the Hurricane Sandy event (16% reduction in annual flood losses). Combining hydrodynamic flood simulations with an economic flood damage cost model, Gourevitch et al. (2020) identified 199 possible floodplain restoration locations as cost-effective measures for flood risk reduction in Lewis Creek watershed, Vermont, USA. The model indicated that the floodplain restoration could reduce the present monetary damages by up to US$400,000 at the cost of only US$75,000. Additionally, the monetary benefits outnumbered the costs by at least 5 to 1 under the maximum rainfall event over 100 years, which is considered as a useful lifetime of an NBS (De Risi et al., 2018).

The Environmental and Energy Study Institute (EESI) estimated the cost of using a sustainable drainage system to manage inland flooding in Los Angeles to be between US$2.8 and US$7.4 billion for green NBS, in contrast to about US$44 billion for grey approaches. Furthermore, application of a new green NBS strategy would cost US$1.2 billion over 25 years, while a commensurate grey approach strategy would cost US$6 billion in Philadelphia (EESI, 2020).

### 5.2 NBS-CBA for droughts

Khogali and Zewdu, 2009a, Khogali and Zewdu, 2009b evaluated the economic performances of NBS such as terraces, earth embankments, irrigated communal vegetable garden and retention pond to mitigate agricultural damages caused by droughts in Sudan. The CBA evaluation criteria B/C ratio (>1) for each intervention with a discount rate (interest rate needed to ascertain the current value of future cash flows) of 10% found the retention pond as the most cost-effective measure (B/C ratio = 2.7), followed by terraces (B/C ratio = 2.5). Mishra and Rai (2014) evaluated the costs and benefits of indigenous soil and water conservation (SWC) practices (e.g., agroforestry, vegetative barriers, terraces, reforestation; stream bank control) to control erosion and increase agricultural production in rural watersheds in Sikkim Himalaya, India. The CBA was performed for selected SWC practices by considering a 10-year period yield, a broad range of NPV, profitability, B/C ratio, payback period, i.e. time period to retrieve the cost of funding, and internal rate of return (IRR), i.e. yearly growth rate of the funding. A 6% discount rate is applied to estimate the NPV. The study found that vegetative barriers and agroforestry were the two most cost-effective practices for reducing soil erosion, increasing crop productivity and income. Terraces were found to be more expensive to be developed than other practices, but they revealed enhanced long-term monetary returns compared to the other SWC practices.

Atampugre (2014) evaluated the net benefits of NBS drought interventions based on NPV and IRR. They found that bench terraces provided the maximum net benefits, followed by contour bunds and Napier grass strips to reduce agricultural risk and increase crop productivity regardless of the crop variety in the Saba sub-catchment of the Upper Tana catchment in Kenya. The study also highlighted that the investment and the associated cost-benefits may not be feasible in the short-term period. Addis et al. (2020) assessed the cost-effectiveness and benefits of SWC measures to decrease soil erosion and enhance soil productivity and crop production in the northern Highlands of Ethiopia. The CBA was performed for numerous measures using NPV and direct market price approach. The results showed that SWC practices reduced the erosion risk by 46.8% and increased soil fertility while reducing the cost of fertilizer between US$3.63 and US$17.97 ha^−1^ year^−1^. The crop yield also increased by 13% to 19.4% ha^−1^ year^−1^, which is translated into economic return values of US$102 and US$140.3 ha^−1^/y^−1^, respectively. SWC practices decreased nutrient exhaustion and significantly enhanced crop production with a NPV of US$477.7 ha^−1^.

### 5.3 NBS-CBA for heatwaves

Natural attributes of a surrounding (greenery, water bodies, etc.) tend to lower down extreme temperatures in a cost-effective manner. For instance, using NPV, EESI (2020) performed the CBA for green gardens, green roofs and tree covers for mitigating heatwaves. The results demonstrated that green roofs could maintain maximum temperatures up to 4.4 °C lower than conventional roofs and decrease citywide temperatures effectively. Also, they can decrease air conditioning costs in premises by up to 75%. The net benefits of green roofs in managing heatwaves are estimated up to US$14 per square foot (US$151 m^−2^) than conventional roofs. Trees and urban parks could buffer and decrease extreme summer temperature by 7 °C with an associated economic benefit of up to US$1.5 to US$3 for every US$1 spent in planting trees, as compared to grey solutions.

The economic benefits derived from the presence of vegetation on walls, roofs, gardens and tram tracks have been examined in various studies. Perini and Rosasco (2013) analysed CBA of green living walls and green façades by considering the human and environmental benefits (e.g. regulating urban temperature and air quality improvement) and the costs throughout their life cycle. The economic sustainability of each NBS intervention was estimated by using three indicators: Payback period, IRR and NPV. The result showed that direct green façades provided positive NPVs between US$11544 and US$36623, and indirect green façades' NPVs varied between US$2504 and US$17878. Of the analysed NBS, the green façades were the most economically sustainable solution to reduce extreme temperature and air pollution. Akabari et al. (2001) demonstrated that the alleviation of the extreme built-up temperature impact with green façades, green roofs and urban trees can decrease the U.S. national energy usage for local climate regulation up to 20%, with a total benefit of more than US$10 billion in energy consumption.

### 5.4 NBS-CBA for landslides

Petrone and Preti (2010) implemented various NBS based on soil bioengineering techniques in the area of Rio Blanco in Nicaragua, including live crib walls, fascine mattresses, palisades and re-vegetation on slopes. The authors compared the costs of the considered NBS between Nicaragua and Italy, also considering the Equal Purchasing Power exchange rate. Depending on the NBS and related involvement of manpower and resources, costs were up to four times lower in Nicaragua. The analysis of the costs also facilitated the consciousness of the economic sustainability of the considered soil bioengineering techniques in developing countries (Petrone and Preti, 2010). Salbego et al. (2015) performed CBA to analyse whether the drainage trenches which promote slope stability by reducing water table could be an economically feasible solution to prevent landslides caused by heavy precipitation in Vicenta province, northeast Italy. The study estimated that the landslides that occurred in 2010 incurred remediation costs of US$367.4 million and infrastructure and building losses of US$1.22 billion. These costs were compared against costs associated with landslide mitigation actions, i.e. post-event actions vs. landslide prevention. The CBA concluded that landslide preventive measures on a single slope scale could yield NPVs of US$21 (C/B-ratio of ~1.75) and on a larger scale a saving potential of 30%, i.e. up to US$ 3.7 million.

Holcombe et al. (2012) also studied the economic feasibility of drainage trenches to mitigate rainfall-triggered landslide damages in unplanned communities in the Caribbean. The resulting C/B-ratio of the analysis was 2.7 to 1 under conservative assumptions. Boonyanuphap (2013) studied the costs and the benefits of vetiver systems from the agricultural perspective to promote land rehabilitation in landslide-destructed cultivated hilly regions in Northern Thailand. The NPV of each rehabilitation method was calculated with four different discount rates. The results showed that all initiatives would likely to diminish the harmful impacts of future landslides events and would provide additional benefits related to the improvement of soil richness, cultivation yield and revenue. If the avoided and/or diminished damages from landslides were monetised, the B/C-ratio of each solution would be most likely to increase.

### 5.5 NBS-CBA for storm surges and coastal erosion

The models for assessment of NBS for storm surges and coastal erosion reviewed herein focused on two types of interventions, i.e., breakwaters and horizontal levees for seawater retention. The integration of numerical models for these types of NBS with CBA has strived to estimate the cost-effectiveness of the NBS, following the calculation of three types of costs through the implementation of LCA approach: (i) total construction or restoration cost of the NBS compared to the costs of deploying the equivalent traditional engineering structure, (ii) ability of the NBS to mitigate/avoid the risk of a particular HMH, being the risk evaluated in monetary/cost terms, and (iii) provision of ecosystem services by the NBS, also quantified in monetary terms. Ferrario et al. (2014) compared the cost efficiency of coral reef restoration to the equivalent traditional breakwater construction which would result in similarly observed wave attenuation. This analysis showed that the traditional breakwater construction costs ranged from US$456 to US$188,817 per metre, while organisational coral reef restoration costs ranged from US$ 20 to US$155,000 per metre. These values are consistent with the recent analysis by Kramer (2016), who concluded that reef restoration was among the most cost-effective methods. Costanza et al. (1997) demonstrated the effectiveness of coastal wetlands in reducing expected damages from hurricane-induced floods. The results showed that a forfeiture of 1 ha of wetland in the model equated to an average US$ 33,000 (median US$ 5000) rise in storm damage costs from particular storms. They also charted the yearly worth of coastal wetlands (at 1 km by 1 km pixels) by state. The value spanned from US$ 250 to US$ 51,000 ha^−1^ yr^−1^, with an average of US$8240 ha^−1^ yr^−1^ (median US$3230 ha^−1^ yr^−1^), an outstandingly larger value than previous estimates. Coastal wetlands in the US were approximated to presently supply US$23.2 billion yr^−1^ in storm defense utilities. They concluded that coastal wetlands furnish “horizontal levees” that are sustained by nature and are a lot more penny-wise than built levees.

### 5.6 Limitations of NBS economic evaluation

The framework of CBA not only formalises the decision-making process but also makes it more transparent for a wider audience, improving the integrity of the process. One can assess the expected outcomes of each NBS alternative as comprehensively as possible by monetising wide-ranging impacts. However, CBA is not a flawless technique and has some limitations. For example, Hansjürgens (2004) identified three main disadvantages of economic evaluation which are reflected by CBA framework: (i) the problems of discounting and compensation, (ii) lack of in-depth understanding of long-term effects, and (iii) problems of substitutability and irreversibilities of essential goods. The weighing up approach and the comparison of human health benefits and monetary gains in CBA has been criticised mainly because of ethical considerations. This objection sustains also in issues related to environmental protection where the economic assessments of projects are feared to be harmful to nature. The rejection of other alternatives based solely on the rankings given after economically weighing all the available choices, constitutes another ground for criticism of CBA because the risks associated with each alternative could not be weighed. The substitutability of various alternatives only depends upon economic considerations. In CBA, future benefits and costs are discounted to the current value which is not justified since the benefits of environmental protection can be experienced after a long-term and are therefore discounted, whereas the costs are taken at its full extent without any kind of discount rate because costs occur in present. The methodology of CBA also has raised many doubts due to lack of a comprehensive multi-sectoral approach and over emphasis on economic aspects of assessment. The CBA and interpretation of its results rely mainly on the expert knowledge of technocrats which makes it undemocratic in the sense that it gives no importance to public opinion. Insufficient and qualitative data with respect to environmental impacts and effects on human health on one hand and strongly-convincing and quantitative economic consequences on the other hand, amplify these shortcomings. There exists some ignorance about the future impacts of a project related to, for example, harmful chemical substances on environment and health. The probability of occurrence of a hazardous event and the scale of its impacts and the damages it may cause, cannot be predicted. This is due to the lack of sufficient data. Collecting large amounts of data for a comprehensive CBA, though not very expensive but it may cause delay in the regulation process but the need for these data could also not be ruled out. The dominance of interests of the economically influential section of society is further strengthened by performing CBA on projects which are likely to affect the health of millions.

Sadik (1978) pointed out six different CBA limitations categories: value judgements, subjectivity, inconsistency, uncertainty, discount rate and income distribution. Uncertainty and discount rate are two distinctive limitations of CBA because NBS have long life cycles with environmental impacts not directly observable from markets. Damart and Roy (2009) found the objectivity of CBA questionable. The methodology of CBA to quantify costs and benefits of a project into monetary equivalents of social and environmental impacts is based on the assumption of arbitrary constructed values and not on actualities that can be transformed straight into pecuniary units. The uncertainty of different CBA components of each alternative is especially problematic in an ex-post CBA where it might be impossible to evaluate probabilities of different outcomes. The challenge is to estimate costs, benefits and impacts of each alternative as comprehensively as possible because the accuracy of components determines the decision maker's confidence in providing recommendations (Sadik, 1978). Many CBA frameworks (e.g., Boardman, 2014; Pearce et al., 2006) require analysts to address the problems regarding uncertainty with methods such as Monte-Carlo simulations, sensitivity analysis or best-worst case analysis. One also faces a problem in comparing present values of costs with possible future benefits by choosing an adequate discount rate. Boardman (2014) mentioned three main unsolved issues which yield varying discount rates: (i) use of present market interest rates to predict weighting of the future consumption, (ii) reflection of the future generation's choices, and (iii) attaching the same value to investment and consumption. Discounting can make severe catastrophes seem trivial on very long time scales of ecological restoration in response to climate change, pollution of radioactive waste and other persistent toxins. Because not all the evaluations can be performed employing modelling solely, surveys and fieldwork are equally important. For example, Chou (2016) conducted an interview of stakeholders comprising 18 questions from six themes, namely availability, actions, public utilities, ecological quality, natural values and flood protection to estimate the qualitative fulfilment of stream restoration. Nevertheless, some of these techniques are only suitable for microscale execution and not for large river basins. Yang et al. (2018a) suggested a ‘relative performance evaluation method, which uses a score to quantify the functioning for all substitutes. This score is estimated as the weighted sum of the scores of discrete indicators. From this discourse, it is evident that simply trying several methods and choosing one by trial and error may not be realistic for intricate systems with a myriad of scenarios and criteria, and an automated optimisation method could help combine the above-mentioned methods.

## 6 Recommendation for future research

Numerous studies have developed and applied numerical models at scattered and urban scale to evaluate the efficiency of specific NBS to manage stormwater/urban flooding (e.g., Elliott and Trowsdale, 2007; Jayasooriya et al., 2016; Zhang and Chui, 2020; Bach et al., 2020). While large scale (landscape or catchment) implementation, monitoring and evaluation of NBS are currently scattered, the majority of information is limited to only flooding and heatwaves in the urban context. Still, it is worth noting that these small-scale implementations have little impact on the large-scale HMHs, such as river flooding, coastline flooding or very intense drought conditions (e.g., propagations) that pose the greatest challenges to communities in terms of water and food supply. This is particularly the case when NBS planned, designed and implemented to address societal challenges, such as climate change and regional groundwater conditions, since both processes impact the performance of NBS on larger scales. Therefore, there is a need to develop multi-scale process-based models to better understand the effectiveness of the pools of NBS on larger spatial scales (e.g., catchment, regional, national) in its broad concepts for disaster risk reduction (DRR) and climate change adaptation and mitigation (Kumar, 2021; Ruangpan et al., 2020). For example, Joyce et al. (2017) advanced a multi-scale modelling technique to evaluate the efficiency of a green-NBS in accordance with the climate change and rising sea levels. They studied the potential influence of green-NBS on groundwater table and flows, but the effect of groundwater on the effectiveness of green-NBS was not evaluated in more detail. Recently, the Urban Biophysical Environments and Technologies Simulator model was developed to consider groundwater table depth in the multi-scale planning and evaluation of green-NBS from street to city scales (e.g., Bach et al., 2020) while the larger-scale impact of NBS is still missing.

Given the highly complicated subsurface geophysical and hydrological conditions and processes, some of HMHs, such as groundwater flooding, drought propagation, and deep-seated landslides require more advanced modelling research approaches to capture their formation, monitoring and evaluating the performance of NBS implemented against them. Modelling techniques, such as spatially distributed numerical models have the ability of processing and forecasting time series while dealing with uncertainty (e.g., Gonzalez-Ollauri and Mickovski, 2017a, Gonzalez-Ollauri and Mickovski, 2017b, Gonzalez-Ollauri and Mickovski, 2017c) and can be very suitable to envisage the performance of pools of NBS at multiple scales and under multiple socio-ecological scenarios. However, numerical models are sometimes not flexible enough to handle multiple spatial and temporal scales. Thus, further research is needed to couple and integrate different spatial-scale process-based models into larger-scale models and also to link with open-access, programming languages and software.

Currently, an abundance of information on NBS is already available and well communicated within the scientific community. For instance, numerous modelling approaches are available that vary in complexity and accuracy, and could help a strategic planning, design, implementation and evaluation of NBS for HMR reductions. However, their application for the implementation and evaluation of NBS effectiveness are hampered by the lack of integration into planning practice and institutional fragmentation (e.g., the lack of systematic mainstreaming between researchers, decision makers and end-users). Hence, implementing NBS requires an overarching integration among researchers (modeller or ecologists), various sectors, policy areas and stakeholders. The proper integration of NBS functions within the fiscal constitution may well support and improve the implementation and evaluation of NBS. Therefore, through the integration of numerical modelling and economic evaluation techniques in collaboration with a variety of stakeholders (e.g., policymakers, landowners, and farmers), it is feasible to implement cost-effective NBS. Furthermore, there is still a lack of fiscal research and guidelines for cost-effective execution of NBS projects and systems that can be employed to encourage new financial and business models for effective implementation of NBS (Ruangpan et al., 2020). Further work is needed for the advancement of databases that integrate functions, benefits and costs of NBS to improve their uptake and upscaling.

There is no single model that integrates ecosystem services and socio-economic impacts for the entire domain of NBS. Despite some recent advances in the efficient simulation of NBS, more research is needed to improve numerical models currently limited by spatial and temporal resolution simulation capacities to develop built-in NBS modules and to integrate the existing numerical models with ecosystem models and CBA tools (multi-model approach). This interdisciplinary modelling demands a strong collaboration and networking among various expertise (e.g., modellers, natural and social scientists) that interconnects the knowledge towards the development of a holistic process-based-ecosystem-CBA model for NBS assessment.

## 7 Conclusions

This work provides an overview of methods used to model the effectiveness of NBS in HMR reduction and to perform its monetary assessment considering all (co)benefits/impacts. To date, such methods to evaluate the broader concepts of NBS, their impacts and benefits are not well communicated within the scientific community and there is an evident lack of holistic appraisal methodology for NBS. This work consolidates the relevant underpinning knowledge and the following conclusions are drawn:

• Since the effectiveness of NBS varies with their typology, functional requirements, types of HMHs and local conditions, preferred modelling techniques rely on practicality and feasibility considerations of the spatial scale of the project, time and funding for data acquisition, model set up, computation and end-user needs. Among the various evaluation approaches, numerical models have typically been more utilised to identify the optimal NBS by evaluating their effectiveness at different spatial scales.
• Among several reviewed numerical models, MIKE-SHE, MODFLOW, SWAT, VELMA, ACRU, SIMGRO, ParFlow-TREES, TELEMAC, and ADCIRC were found to be capable of assessing NBS optimal allocation and effectiveness against HMHs at the catchment scale. However, they are data intensive, limited in spatial and temporal resolutions and cannot simulate detailed geometry for designing features of NBS on scattered scale. Other numerical models, such as SWMM, LISFLOOD-FP, ENVI-met are accurate and flexible but are unable to evaluate large scale NBS designing and planning.
• Hydrological (MIKE-SHE, SWAT) and hydraulic (HEC-RAS, LISFLOOD-FP, Flood Modeller) models are the extensively utilised numerical models to evaluate the efficiency of wetlands, ponds, weirs, trees and green roofs in reducing flood risks and associated damages from micro to large scales. MIKE-SHE, SWAT, HEC-RAS, and MODFLOW are spatially-distributed widely used models at the watershed scale and require copious spatial data which may not always be available, and their spatio-temporal resolutions are usually coarse. The grid size in these models exceeds the normal size of green NBS and they often cannot evaluate the effectiveness of individual green NBS in reducing surface runoff. They also do not support the evaluation of SUDS, which hinders their applications in urban regions. Conversely, SWMM, LISFLOOD-FP and Flood Modeller can be applied at a local scale, even for the individual NBS attributes.
• Process-based models such as ParFlow-TREES, ACRU and SIMGRO are mostly used to evaluate the efficiency of NBS implemented against drought risk such as trees or thicket vegetation. Droughts typically occur at the catchment scales or larger areas and these models are capable of evaluating the performances of NBS implemented for drought risks at these scales.
• The micrometeorological model ENVI-met and *meso*scale model WRF have been widely utilised to evaluate the efficiency of NBS (green roofs/walls, urban parks, water bodies etc.) at the micro and meso scales. ENVI-met allows more flexibility and details in the simulation of green NBS than WRF, although both models have their own application niche, mainly determined by the project scale.
• To date, different numerical models have been adopted to simulate the efficiency of specific NBS against landslide risks. Of them, PLAXIS, FUNWAVE-TVD, SSHV-2D, tRIBS-VEGGIE and BROOK90 models are widely used and especially suitable to assess the root reinforcement of shrubs, trees, forest, and grasslands for slope stabilisation.
• It is challenging to evaluate the storm surges and coastal erosion defense of NBS due to the widely variable trajectories, severities, frequencies, and effects of storms. The reviewed numerical models SWAN, TELEMAC, XBeach, ADCIR are the most extensively used to evaluate coastal NBS (e.g., mangroves, sea grass, coral reefs, saltmarsh) against storm surge and coastal erosion risks. The 3D versions of TELEMAC and ADCIRC models are the most thorough at simulating the complex phenomena that drive the sea waves' propagation and anomalies.
• The limitations associated with numerical models are; their input data requirements, spatial and temporal discretisation (grids or mesh, time), computational difficulties (time and complexity) and lack of modules/packages that integrate all ecosystem services and their feedback loops or interactions among the numerical models and ecosystem functions and services.
• CBA and its different variants are widely used to assess the economic efficiency of projects based on evaluation criteria such as NPVs, B/C ratio and IRR. CBA studies comparing NBS against grey solutions for HMRs reduction are still scarce. However, the existing studies clearly indicate that nature-based interventions for HMHs such as flooding (green roofs, wetlands and forest), droughts (vegetative barriers, agroforestry, bench terrace, grass strips, and contour bunds), heatwaves (green façades, green roofs and urban trees), landslides (drainage trenches, vegetation roots) and, storm surges and coastal erosion (coral reef restoration and coastal wetlands) have great economic benefits with a positive NPVs, IRR and B/C ratio (>1), compared to the grey approaches.
• CBA is advantageous in comparing different alternatives, subjected to heterogenous criteria, as it normalises all their impacts and benefits in monetary terms. It provides a comparison framework and helps make rational decisions in complex situations. However, monetisation of intangible benefits such as biodiversity, aesthetic values, mental and physical health is difficult and subjective. The CBA method has problems of uncertainty, discounting, substitutability and compensation for long-term effects of NBS.

## CRediT authorship contribution statement

PK: Conceptualisation, Supervision, Project Administration, Writing - Original Draft, Writing - Review & Editing; SD, JS, BM, NR and PK: Methodology, Data Analysis (Figures, Tables), Writing - Original Draft, including 1, 2, 3.1, 3.2, 3.3, 4, 5.5, 3, 5, 6, 7, 5.3, 5.6; 6; BB, AB, FP, SS, PB, JP & SM: Writing - review and editing (3.1, 4, 5.1); NC, ML and CS: Writing - review and editing (3.2, 4); SBM: Writing - reviewing & editing (3.5, 4, 5.5); JP, MR, MM and TZ: Writing - review & editing (3.4, 5.4); JJ & HT: Writing - reviewing & editing (5, 5.1, 5.2, 5.4, 5.6). LSL, AB, MM, GG, MS: Writing - reviewing and editing the manuscript. All authors: Writing - reviewing & editing. The names of co-authors appear in alphabetical order according to their surname, after the core writing team.

## Declaration of competing interest

The authors declare no conflict of interest.

## Appendix A Supplementary data

Supplementary material Image 1
