# Supplementary material for: Nature-based solutions efficiency evaluation against natural hazards: Modelling methods, advantages and limitations
Source: Sci Total Environ. 2021 Aug 25;784:147058. doi: 10.1016/j.scitotenv.2021.147058 (PMC8192688; doi:10.1016/j.scitotenv.2021.147058)
Supplement: Supplementary file 1 — Supplementary material [file mmc1.docx]

**Supplementary Information (SI)**

*for*

**Nature-based solutions efficiency evaluation against natural hazards: modelling methods, advantages and limitations**

Prashant Kumar^a,b,^^[[1]](#footnote-1)^, Sisay E. Debele^a^, Jeetendra Sahani^a^, Nidhi Rawat^a^, Belen Marti-Cardona^a^, Silvia Maria Alfieri^c^, Bidroha Basu^b,d^, Arunima Sarkar Basu^d^, Paul Bowyer^e^, Nikos Charizopoulos^f,g^, Glauco Gallotti^h^, Juvonen Jaakko^i^, Laura S. Leo^n^, Michael Loupis^j,k^, Massimo Menenti^c,l^, Slobodan B. Mickovski^m^, Seung-Jae Mun^e^, Alejandro Gonzalez-Ollauri^m^, Jan Pfeiffer^n^, Francesco Pilla^d^, Julius Pröll^e^, Martin Rutzinger^o^, Marco Antonio Santo^h^, Srikanta Sannigrahi^d^, Christos Spyrou^j,p^, Heikki Tuomenvirta^i^, Thomas Zieher^n^

^a^*Global Centre for Clean Air Research (GCARE), Department of Civil and Environmental Engineering, Faculty of Engineering and Physical Sciences, University of Surrey, Guildford GU2 7XH, United Kingdom*

*^b^Department of Civil, Structural & Environmental Engineering, School of Engineering, Trinity College Dublin, Dublin, Ireland*

*^c^Department of Geoscience and Remote Sensing, Delft University of Technology, Delft, The Netherlands*

*^d^School of Architecture, Planning and Environmental Policy, University College Dublin, Dublin, Ireland*

*^e^Climate Service Center Germany (GERICS), Helmholtz-Zentrum Geesthacht, Hamburg, Germany*

*^f^Agricultural University of Athens, Laboratory of Mineralogy-Geology, Iera Odos 75, 118 55 Athens, Greece*

*^g^Region of Sterea Ellada, Kalivion 2, 351 32, Lamia, Greece*

*^h^Department of Physics and Astronomy (DIFA), University of Bologna, Bologna, Italy*

*^i^Finnish Meteorological Institute, Erik Palménin Aukio 1, 00560 Helsinki, Finland*

*^j^Innovative Technolo^[[2]](#footnote-2)^gies Center S.A., Alketou Str. 25, 11633 Athens, Greece*

*^k^National & Kapodistrian University of Athens, Psachna 34400, Greece*

*^l^Aerospace Information Research Institute, Chinese Academy of Sciences, Beijing, China*

*^m^The Built Environment Asset Management Research Centre, Glasgow Caledonian University, G4 0BA Glasgow, Scotland, United Kingdom*

*^n^Institute for Interdisciplinary Mountain Research, Austrian Academy of Sciences,*

*Innsbruck, Austria*

*^o^Institute of Geography, University of Innsbruck, Innsbruck Austria*

*^p^lInstitute for Astronomy, Astrophysics, Space Applications and Remote Sensing (IAASARS), National Observatory of Athens,15236 Athens, Greece*

**
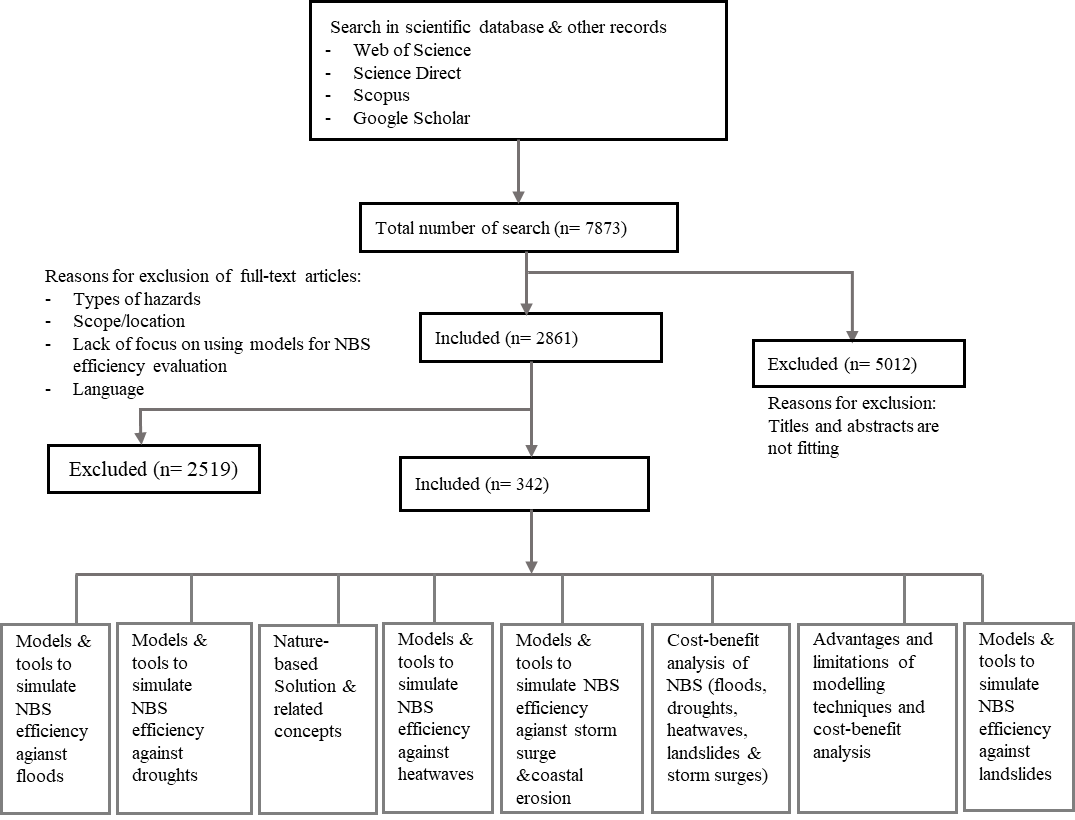
**

**Figure S1**. Schematic representation of a systematic literature review (SLR) and reasons for exclusion.

**Table S1.** Summary of past review papers on the assessment of natural hazards, management strategies and their efficiency evaluation methods.

| Article focus and key finding(s) | | Author (year) |
| --- | --- | --- |
| - Presented a framework for application of cloud computing (CC) for advancing geospatial modelling in natural disaster management - The framework was extended to overcome the challenge of CC before, during and after the occurrence of disaster by including evolving technologies, e.g. IoT network, fog and edge computing. | Ujjwal et al. (2019) | |
| - Presented a conceptual framework and set of indicators for vulnerability and risk assessment in the context of NBS. - A list of 135 indicators considering multi-benefits of NBS was identified that can be adapted for other natural hazards DRR. | Shah et al. (2020) | |
| - Presented methods used to evaluate three HMRs (flood, drought and heatwaves) and their management via NBS. - The capability of models to evaluate the HMRs depend on the input variables, data quality, spatial and temporal resolutions, and the selected management strategy, e.g. NBS. | Sahani et al. (2019) | |
| - Presented a comprehensive categorization for review of decisions for natural DRR, which includes scoping, analysis methodologies, problem drafting, organizational and user interplay with the system, user involvement, monitoring, and assessment. - The review indicated that most of the work has been done on identification risk prone areas and the associated financial damages, lacking research on validating risk management measures concerning anticipated risk. | Newman et al. (2017) | |
| - Presented current approach and indices for evaluating water-management NBS and water systems for natural and artificial components, and barriers to current NBS policies. - They found that the co-benefits of water-management NBS have not been properly addressed. So, a complete water system evaluation is needed by combining current methods, models and indicators. | Nika et al. (2020) | |
| - Reviewed the impact assessment of NBS in Europe and identified four theoretical challenges and three practical gaps. - Derived principles for impact assessment frameworks providing recommendations for NBS monitoring and assessment for evidence building. | Dumitru et al. (2020) | |
| - Reviewed five modelling techniques (agent-based, event and fault trees, system dynamic, Bayesian networks and hybrid models) for their prospect use in multi-risk assessment and CCA in mountain environments. - The study showed inadequacy of the chosen approaches in tackling the risks in highland areas. Hybrid and system dynamic models were proposed to simulate climate change impacts on multi-risks. | Terzi et al. (2019) | |
| - Reviewed the role of NBS in providing ecological services and adaptation to extreme coastal storms. - It was concluded that the benefits of NBS vary with location. Rigorous strength evaluation is needed before selecting any particular NBS at a specific-site. | Saleh et al. (2016) | |
| - Identified the problems of executing infiltration-based green NBS in shallow groundwater areas and evaluated the methods to assess these NBS. - Numerical techniques were found to be better than laboratory or in-situ measurement and remote sensing for sub-surface flows tracing and multivariable analysis. Tracer studies and use of variably saturated, multi-scale, artificially-intelligent and improved hydrologic models were recommended for further research. | Zhang and Chui (2019) | |
| - Reviewed existing multi-risk evaluation concepts to develop a multi-risk assessment approach in climate change scenario. - Selection of dynamic hazard, exposure and vulnerability metrics with collaboration of cross-sectoral expertise was proposed. | Gallina et al. (2016) | |
| - Consolidated information to measure hazard interrelationships (triggering, change condition, compound, independence and mutually exclusive) considering 14 natural hazards and 19 modelling methods (stochastic, empirical or mechanistic). - 24 different interrelationships were shown using two matrices (cascading and compound hazards). Suitable modelling and physical information of hazard(s) are effective for hazard interaction evaluation. | Tilloy et al. (2019) | |
| - Provided a taxonomy, a method and a research programme for evaluating the economic value of disasters. - They highlighted- (i) the application of the latest platform (e.g. crowdsourcing and social media) and predetermined economic value indicators can enhance response time for disaster evaluation, (ii) the use of economic methods and cost of slow-onset disaster have been very less addressed in literature. | Eckhardt et al. (2019) | |
| - Reviewed different economic methods for forested watershed management decisions. - The review showed that studies associating forest maintenance with watershed benefits in financial decision-support are very limited. The challenges are sparse, complex and confidential geo-referenced data, its cost and high demands for modern spatial econometric models. | Ovando et al. (2019) | |
| - Presented an analytical framework of combining plant-wide models to life cycle assessment to find the best wastewater treatment scheme assessing effluent quality, greenhouse gas emission, running cost and aggregate ecological impact. - They concluded that the application of plant-wide models linked with LCA can attain effective results with least environmental ill-effects. | Nguyen et al. (2020) | |
| - Reviewed different tools for disaster monitoring, detection, prediction and management, particularly in the context of landslides, forest fires and earthquakes. - They recommended extensive radio resource maintenance methods driven by artificial intelligence or machine learning to allow automated real-time operations for future studies. | Khan et al. (2020) | |
| - Reviewed hydrodynamic, simple conceptual and empirical approaches for delineating flood risk along with their strengths and weaknesses. - Suggestions were provided for choosing the most appropriate method for addressing realistic flood associated problems, considering the particular outputs needed for the modelling objectives, the data availability and computational difficulties. Multi-model and multidisciplinary perspectives were suggested for further research. | Teng et al. (2017) | |
| - Presented the implementation steps of NBS, using Open-Air Laboratories as a user-centric approach. - They highlighted the importance of stakeholder involvement throughout the life cycle of NBS projects, its monitoring and evaluation, and interaction among scientists, policy makers and end users for comprehensive management of HMHs. | Kumar et al. (2020) | |
| - Reviewed NBS intervention scales (small and large) focusing on four HMHs (floods, droughts, storm surges and landslides), the existing methods for NBS appraisal and the major socio-economic factors affecting the implementation process of NBS. - They highlighted that the performance, benefits, and acceptance of NBS are dependent on the implementation objective, local context, and cultural settings. | Ruangpan et al. (2020) | |

**Table S2.** Keyword search results from different scientific databases.

| Keyword | Web of Science | Science Direct | Scopus | Google Scholar |
| --- | --- | --- | --- | --- |
| “Models & tools to simulate NBS efficiency against floods” | 400 | 800 | 200 | 82 |
| “Nature-based Solution & related concepts” | 300 | 400 | 150 | 80 |
| “Models & tools to simulate NBS efficiency against droughts” | 250 | 350 | 125 | 75 |
| “Models & tools to simulate NBS efficiency against storm surge & coastal erosion” | 200 | 150 | 100 | 63 |
| “Models & tools to simulate NBS efficiency against heatwaves” | 300 | 100 | 120 | 68 |
| “Cost-benefit analysis of NBS (floods, droughts, heatwaves, landslide & storm surge)” | 160 | 125 | 100 | 70 |
| “Climate change” | 300 | 200 | 100 | 15 |
| “Models & tools to simulate NBS efficiency against landslides” | 100 | 80 | 60 | 5 |
| “Natural hazards” | 300 | 500 | 50 | 4 |
| “Modelling methods to evaluate nature-based solutions against natural hazards” | 200 | 105 | 78 | 3 |
| “Modelling methods for green and blue approaches” | 60 |  | 60 | 5 |
| “A review on assessment methodologies for natural hazards” | 70 | 45 | 150 | 8 |
| “Modelling and evaluating cost of benefit nature solutions” | 40 | 60 | 100 | 7 |
| “Evaluation methods green infrastructure efficiency against natural hazards” | 30 | 40 | 70 | 10 |
| “Advantage and limitation of numerical models in evaluation the efficiency of NBS” | 70 | 100 | 37 | 5 |
| Total | 2780 | 3055 | 1500 | 500 |

**References**

Dumitru, A., Frantzeskaki, N., Collier, M. (2020). Identifying principles for the design of robust impact evaluation frameworks for nature-based solutions in cities. Environmental Science & Policy 112, 107-116.

Eckhardt, D., Leiras, A., Thomé, A.M.T. (2019). Systematic literature review of methodologies for assessing the costs of disasters. International Journal of Disaster Risk Reduction 33, 398-416.

Gallina, V., Torresan, S., Critto, A., Sperotto, A., Glade, T., Marcomini, A. (2016). A review of multi-risk methodologies for natural hazards: Consequences and challenges for a climate change impact assessment. Journal of Environmental Management 168, 123-132.

Khan, A., Gupta, S. and Gupta, S.K. (2020). Multi-hazard disaster studies: monitoring, detection, recovery, and management, based on emerging technologies and optimal techniques. International Journal of Disaster Risk Reduction 47, 101642.

Kumar, P., Debele, S. E., Sahani, J., Aragão, L., Barisani, F., Basu, B., Bucchignani, E., Charizopoulos, N., Di Sabatino, S., Domeneghetti, A., Edo, A. S., Finér, L., Gallotti, G., Juch, S., Leo, L. S., Loupis, M., Mickovski, S. B., Panga, D., Pavlova, I., Pilla, F., Prats, A. L., Renaud, F. G., Rutzinger, M., Sarkar, A., Shah, M. A. R., Soini, K., Stefanopoulou, M., Toth, E., Ukonmaanaho, L., Vranic, S., Zieher, T. (2020). [Towards an operationalisation of nature-based solutions for natural hazards.](https://doi.org/10.1016/j.scitotenv.2020.138855) Science of The Total Environment 731, 138855.

Nguyen, T.K.L., Ngo, H.H., Guo, W.S., Chang, S.W., Nguyen, D.D., Nghiem, L.D., Nguyen, T.V. (2020). A critical review on life cycle assessment and plant-wide models towards emission control strategies for greenhouse gas from wastewater treatment plants. Journal of Environmental Management 264, 110440.

Nika, C.E., Gusmaroli, L., Ghafourian, M., Atanasova, N., Buttiglieri, G., Katsou, E (2020). Nature-based solutions as enablers of circularity in water systems: A review on assessment methodologies, tools and indicators. Water research 183, 115988.

Newman, J.P., Maier, H.R., Riddell, G.A., Zecchin, A.C., Daniell, J.E., Schaefer, A.M., van Delden, H., Khazai, B., O'Flaherty, M.J., Newland, C.P. (2017). Review of literature on decision support systems for natural hazard risk reduction: Current status and future research directions. Environmental Modelling and Software 96, 378-409.

Ovando, P., Brouwer, R. (2019). A review of economic approaches modeling the complex interactions between forest management and watershed services. Forest Policy and Economics 100, 164-176.

Sahani, J., Kumar, P., Debele, S., Spyrou, C., Loupis, M., Aragão, L., Porcù, F., Shah, M.A.R., Di Sabatino, S. (2019). Hydro-meteorological risk assessment methods and management by nature-based solutions. Science of the Total Environment 696, 133936.

Saleh, F., Weinstein, M.P. (2016). The role of nature-based infrastructure (NBI) in coastal resiliency planning: A literature review. Journal of Environmental Management 183,1088-1098.

Shah, M.A.R., Renaud, F.G., Anderson, C.C., Wild, A., Domeneghetti, A., Polderman, A., Votsis, A., Pulvirenti, B., Basu, B., Thomson, C., Panga, D. et al. (2020). A review of hydro-meteorological hazard, vulnerability, and risk assessment frameworks and indicators in the context of nature-based solutions. *I*nternational Journal of Disaster Risk Reduction 50, 101728.

Teng, J., Jakeman, A.J., Vaze, J., Croke, B.F., Dutta, D., Kim, S. (2017). Flood inundation modelling: A review of methods, recent advances and uncertainty analysis. Environmental Modelling & Software 90, 201-216.

Terzi, S., Torresan, S., Schneiderbauer, S., Critto, A., Zebisch, M., Marcomini, A. (2019). Multi-risk assessment in mountain regions: A review of modelling approaches for climate change adaptation. Journal of Environmental Management 232, 759-771.

Tilloy, A., Malamud, B.D., Winter, H., Joly-Laugel, A. (2019). A review of quantification methodologies for multi-hazard interrelationships. Earth-Science Reviews 196, 102881.

Ujjwal, K.C., Garg, S., Hilton, J., Aryal, J., Forbes-Smith, N. (2019). Cloud Computing in natural hazard modeling systems: Current research trends and future directions. International Journal of Disaster Risk Reduction 38, 101188.

Zhang, K., Chui, T.F.M. (2019). A review on implementing infiltration-based green infrastructure in shallow groundwater environments: Challenges, approaches, and progress. Journal of Hydrology 579, 124089.

1. Corresponding author. Address as above. Email [p.kumar@surrey.ac.uk](mailto:p.kumar@surrey.ac.uk), [Prashant.Kumar@cantab.net](mailto:Prashant.Kumar@cantab.net) (Prashant Kumar) [↑](#footnote-ref-1)
2. Corresponding author. Address as above. E-mail addresses: P.Kumar@surrey.ac.uk, Prashant.Kumar@cantab.net [↑](#footnote-ref-2)
